# Supplementary material for: A Bayesian neural ordinary differential equations framework to study the effects of chemical mixtures on survival
Source: PLoS Comput Biol. 2025 Nov 10;21(11):e1013681. doi: 10.1371/journal.pcbi.1013681 (PMC12674509; doi:10.1371/journal.pcbi.1013681)
Supplement: S1 Appendix — (PDF) [file pcbi.1013681.s001.pdf]

Supporting Information for A Bayesian Neural Ordinary Differential Equations Framework to Study the Effects of Chemical Mixtures on Survival

November 3, 2025

Virgile BAUDROT[1*],Nina CEDERGREEN[2],Thomas KLEIBER[1],André GERGS[3], Sandrine CHARLES[4]

1 Qonfluens, Montpellier, France

2 University of Copenhagen, Copenhagen, Denmark

3 Bayer AG, Monheim, Germany

4 Université Claude Bernard Lyon 1, Villeurbanne, France

1 Summary of the four datasets

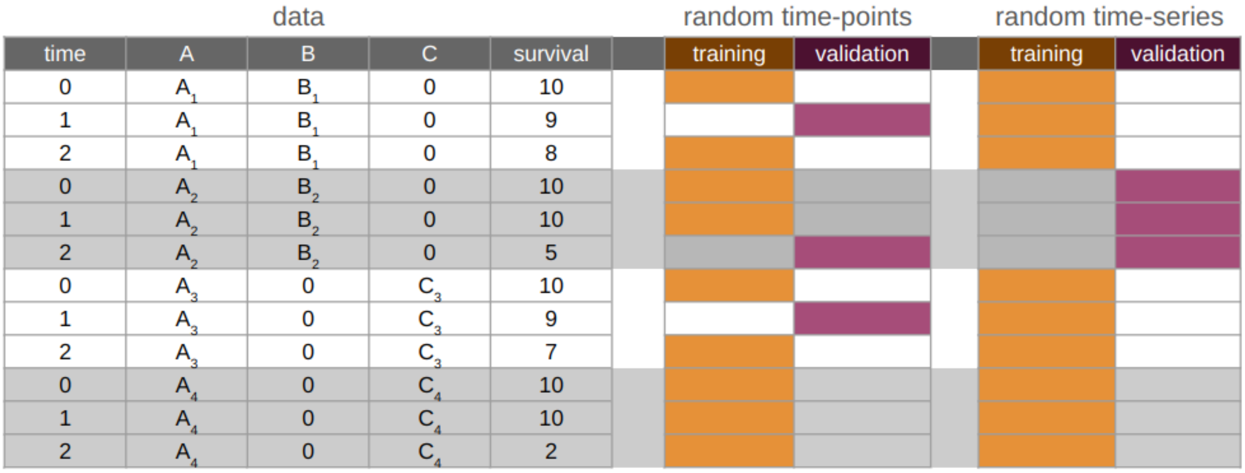

Figure A: Visualization of dataset splitting for two different binary mixtures (AB and AC), both sharing compound A. In orange, the training subset is shown, where the model is fitted, and parameters are estimated. In purple, the validation subset is displayed, where model predictions are compared for validation. The “random time-points” columns represent a random selection of 90% of time points across the entire dataset for calibration, with the remaining 10% used for validation. The “random time-series” columns depict the selection of 90% of time series for calibration and 10% for validation, respectively.

	time	A	B	C	survival	training	validation
AB	0	A_1	B_1	0	10		
	1	A_1	B_1	0	9		
	2	A_1	B_1	0	8		
	0	A_2	B_2	0	10		
	1	A_2	B_2	0	10		
	2	A_2	B_2	0	5		
AC	0	A_3	0	C_3	10		
	1	A_3	0	C_3	9		
	2	A_3	0	C_3	7		
	0	A_4	0	C_4	10		
	1	A_4	0	C_4	10		
	2	A_4	0	C_4	2		
BC	0	0	B_5	C_5	10		
	1	0	B_5	C_5	5		
	2	0	B_5	C_5	1		
	0	0	B_6	C_6	10		
	1	0	B_6	C_6	9		
	2	0	B_6	C_6	3		

Figure B: Illustration of the selection scheme used for validation on uncalibrated mixtures. Across the entire dataset, all mixtures containing a specific pair of active ingredients (e.g., AB) are excluded from the calibration dataset and reserved exclusively for validation.

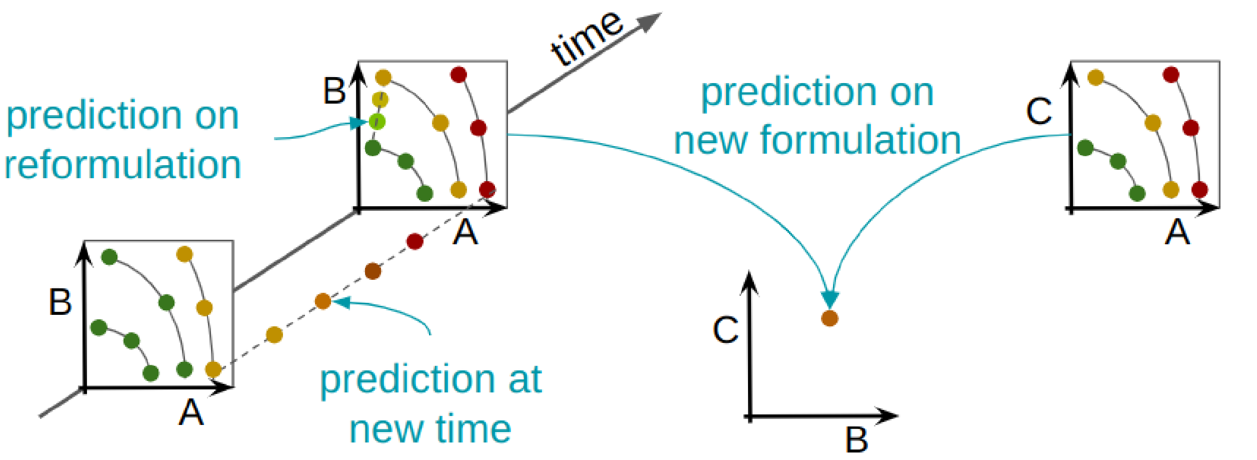

Figure C: The figure illustrates three prediction types: (1) predictions at a new time point with the identical mixture; (2) reformulation predictions (same active ingredients but in different proportions); and (3) predictions for not yet tested newly composed mixtures of tested active ingredients.

2 Information about OECD 203 guidelines

The OECD Test Guideline 203 describes the Fish Acute Toxicity Test, where fish are exposed to a chemical for 96 hours under static, semi-static, or flow-through conditions. The purpose is to determine mortality rates and visible abnormalities, and to estimate the LC50 (the concentration lethal to 50% of the fish). The test requires at least five concentrations in a geometric series and a minimum of seven fish per concentration, with exposure conditions carefully controlled for oxygen levels, temperature, light, and water quality. The guideline also allows for the optional collection of biological samples (e.g., tissues for omics studies) for further mechanistic investigations.

Data collection focuses on both quantitative and qualitative endpoints. Mortality is the primary endpoint, but sublethal effects such as abnormal swimming, pigmentation changes, respiratory distress, or loss of equilibrium are systematically recorded. Observations are made multiple times during the first 24 hours (e.g., at 2–6 hours and 24 hours) and then twice daily until 96 hours. Water parameters (oxygen, pH, temperature, salinity if relevant) are measured daily, while chemical concentrations are verified analytically at defined intervals to confirm exposure levels. Profiles of exposure concentration over time are carefully tracked, since maintaining concentrations close to nominal ($\geq$80%) is critical for test validity.

The guideline stresses both experimental rigor and animal welfare. Fish must be acclimatized for at least 9 days before testing, healthy, and from the same cohort. Controls (dilution water and/or solvent controls) are required. Dead fish are removed promptly, and humane endpoints are encouraged if sublethal signs predict imminent death. At the end of the test, surviving fish are euthanized, and optional cryopreservation of tissues is recommended for omics analyses. Data are reported as LC50 values at different time steps (24, 48, 72, 96 h), concentration–response curves, and detailed records of observed abnormalities.

2.1 Dataset 1

Dataset 1 is made of 244 time-series covering 21 mixtures, given 1482 survival data points.

The active ingredients are: bixafen, fluopyram, prothioconazole, spiroxamine, tebuconazole and trifloxystrobin.

And the 21 mixtures are given as follows:

2.2 Dataset 2

Dataset 2 is made of 120 time-series covering 9 mixtures, given 662 survival data points.

The active ingredients are: deltamethrin, flupyradifurone, spiromesifen, tralomethrin and triazophos.

2.3 Dataset 3

Dataset 3 is made of 130 time-series covering 11 mixtures, given 727 survival data points.

The active ingredients are: beta-cyfluthrin, clothianidin, cyfluthrin, imidacloprid, thiacloprid and thiodicarb.

2.4 Dataset 4

Dataset 4 is made of 126 time-series covering 12 mixtures, given 758 survival data points.

The active ingrediens are: aclonifen, diflufenican, flufenacet, flurtamone and metribuzin.

Mixtures	Number of time series
bixafen	13
bixafen, fluopyram	6
bixafen, fluopyram, prothioconazole	6
bixafen, prothioconazole	6
bixafen, prothioconazole, spiroxamine	6
bixafen, prothioconazole, tebuconazole	6
bixafen, tebuconazole	6
fluopyram	6
fluopyram, spiroxamine	6
fluopyram, tebuconazole	6
fluopyram, trifloxystrobin	18
prothioconazole	25
prothioconazole, spiroxamine	6
prothioconazole, spiroxamine, tebuconazole	6
prothioconazole, spiroxamine, trifloxystrobin	6
prothioconazole, tebuconazole	18
prothioconazole, trifloxystrobin	12
spiroxamine	22
tebuconazole	22
tebuconazole, trifloxystrobin	12
trifloxystrobin	30

Table A: Dataset 1 mixtures.

Mixtures	Pairwise availability of data
bixafen, fluopyram	data
bixafen, prothioconazole	data
bixafen, spiroxamine	no data
bixafen, tebuconazole	data
bixafen, trifloxystrobin	no data
fluopyram, prothioconazole	no data
fluopyram, spiroxamine	data
fluopyram, tebuconazole	no data
fluopyram, trifloxystrobin	data
prothioconazole, spiroxamine	data
prothioconazole, tebuconazole	data
prothioconazole, trifloxystrobin	data
spiroxamine, tebuconazole	no data
spiroxamine, trifloxystrobin	no data
tebuconazole, trifloxystrobin	data

Table B: Pairwise for dataset 1.

Mixtures	Number of time series
deltamethrin	39
deltamethrin, flupyradifurone	12
deltamethrin, tralomethrin	12
deltamethrin, triazophos	8
flupyradifurone	7
flupyradifurone, spiromesifen	7
spiromesifen	13
tralomethrin	14
triazophos	8

Table C: Dataset 2 mixture.

Mixture	Pairwise availability of data
deltamethrin, flupyradifurone	data
deltamethrin, spiromesifen	no data
deltamethrin, tralomethrin	data
deltamethrin, triazophos	data
flupyradifurone, spiromesifen	data
flupyradifurone, tralomethrin	no data
flupyradifurone, triazophos	no data
spiromesifen, tralomethrin	no data
spiromesifen, triazophos	no data
tralomethrin, triazophos	no data

Table D: Pairwise for dataset 2.

Mixtures	Number of time series
beta-cyfluthrin	25
beta-cyfluthrin, clothianidin	6
beta-cyfluthrin, thiacloprid	6
clothianidin	7
clothianidin, imidacloprid, thiodicarb	6
cyfluthrin	13
cyfluthrin, imidacloprid	7
imidacloprid	21
imidacloprid, thiodicarb	19
thiacloprid	7
thiodicarb	13

Table E: Dataset 3 mixture.

Mixtures	Pairwise availability of data
beta-cyfluthrin, clothianidin	data
beta-cyfluthrin, cyfluthrin	no data
beta-cyfluthrin, imidacloprid	no data
beta-cyfluthrin, thiacloprid	data
beta-cyfluthrin, thiodicarb	no data
clothianidin, cyfluthrin	no data
clothianidin, imidacloprid	no data
clothianidin, thiacloprid	no data
clothianidin, thiodicarb	no data
cyfluthrin, imidacloprid	data
cyfluthrin, thiacloprid	no data
cyfluthrin, thiodicarb	no data
imidacloprid, thiacloprid	no data
imidacloprid, thiodicarb	data
thiacloprid, thiodicarb	no data

Table F: Pairwise for dataset 3.

Mixtures	Number of time series
aclonifen	25
aclonifen, diflufenican	6
aclonifen, diflufenican, flufenacet	7
aclonifen, flufenacet	6
aclonifen, flurtamone	6
diflufenican	12
diflufenican, flufenacet	14
diflufenican, flufenacet, flurtamone	6
diflufenican, metribuzin	6
flufenacet	21
flurtamone	7
metribuzin	10

Table G: Dataset 4 mixture.

Mixtures	Pairwise availability of data
aclonifen diflufenican	data
aclonifen flufenacet	data
aclonifen flurtamone	data
aclonifen metribuzin	no data
diflufenican flufenacet	data
diflufenican flurtamone	no data
diflufenican metribuzin	data
flufenacet flurtamone	no data
flufenacet metribuzin	no data
flurtamone metribuzin	no data

Table H: Pairwise for dataset 4.

3 Checking potential effect of co-formulants

In order to make sure that co-formulant do not have an effect on the survival, we compared the LC50 predicted with the formulation with the one predicted with active ingredients. We did it on single compound formulation which share the same co-formulant are those used in formulation with mixture of several active ingredients. No additional effects of co-formulant were detected.

In practice, using for instance a time series of a study on spiroxamine formulation at 97.8. One one side, we fit the data considering the formulation concentration and then compute the LC50 (which was 17.516 [15.817, 18.245]) and one the other side we fit data considering the active ingredient concentration only, where we obtain the LC50 (17.121 [15.541, 17.844]). As a measure of discrepancy, we compared the ratio between the two LC50 with the ratio of active ingredients in the formulation. In this example of spiroxamine, it corresponds to LC50 active ingredient / LC 50 formulation = 17.121 / 17.516 with 97.8), and the difference gives -0.00055, leading to consider no effect of the co-formulant in this situation. We did the same procedure for all sets of time series with a single active ingredient, and the results are given in the graphics here-after. This difference never exceeds 0.015.

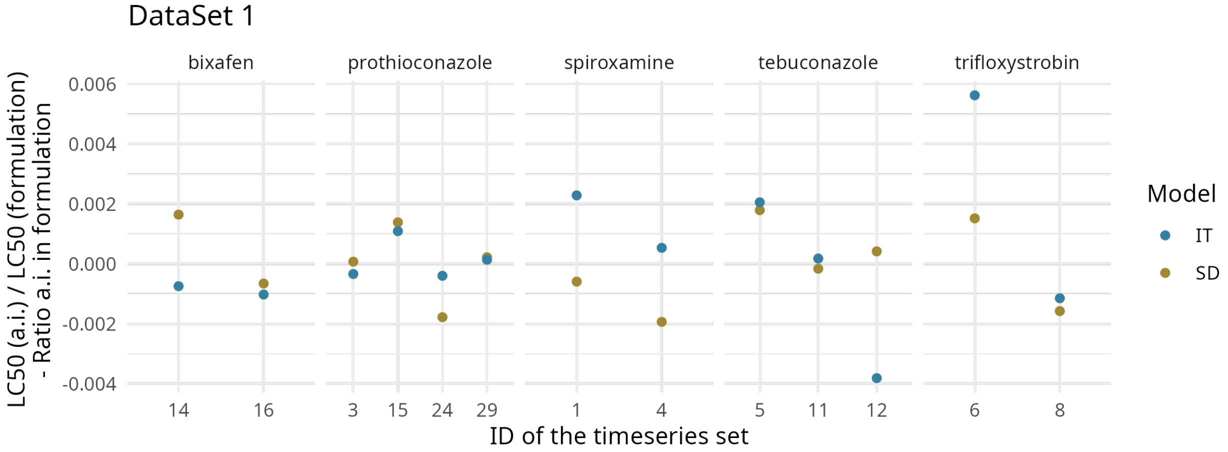

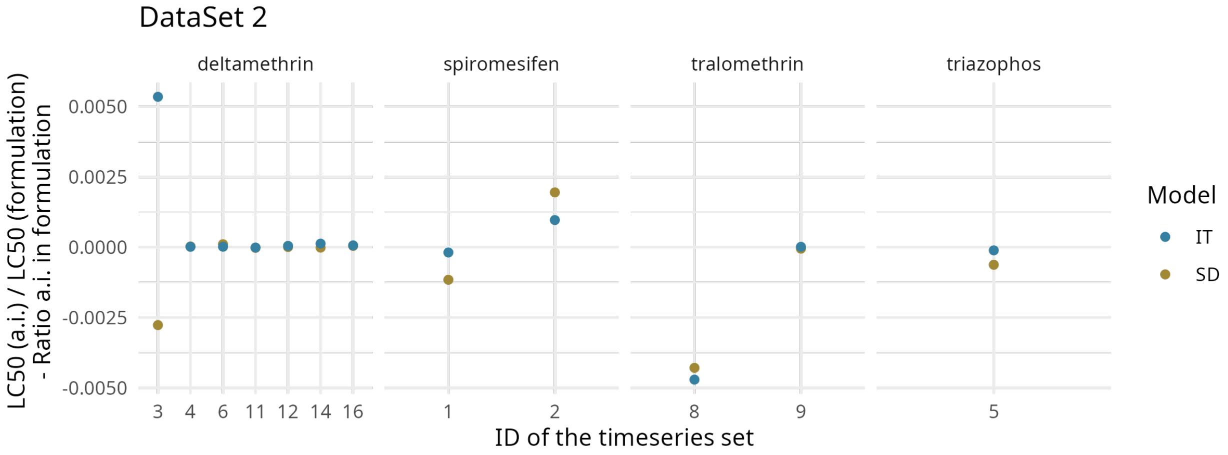

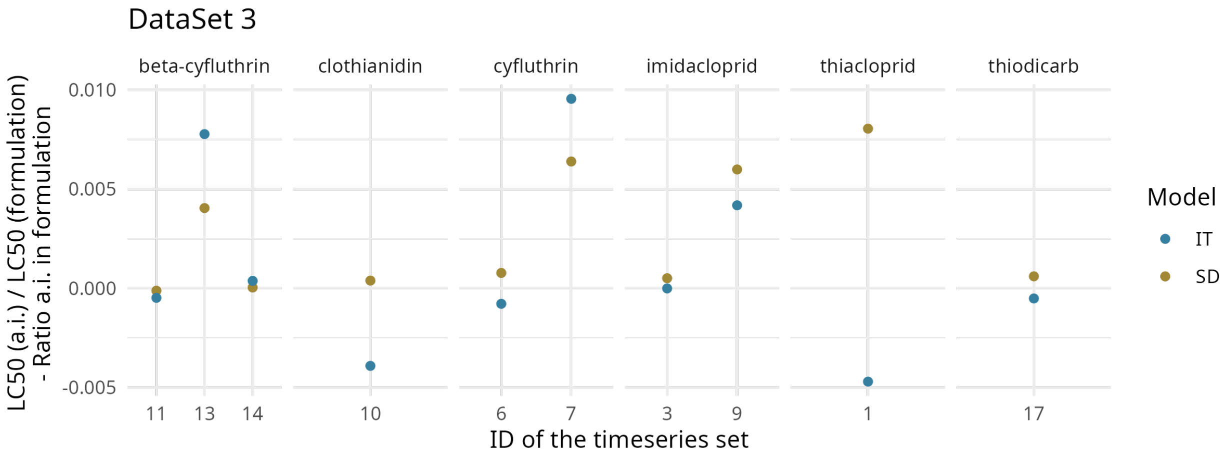
DataSet 3
beta-cyfluthrin
clothianidin
cyfluthrin
imidacloprid
thiacloprid
thiodicarb
LC50 (a.i.) / LC50 (formulation) - Ratio a.i. in formulation
0.010
0.005
0.000
-0.005
11
13
14
10
6
7
3
9
1
17
ID of the timeseries set
Model
IT
SD

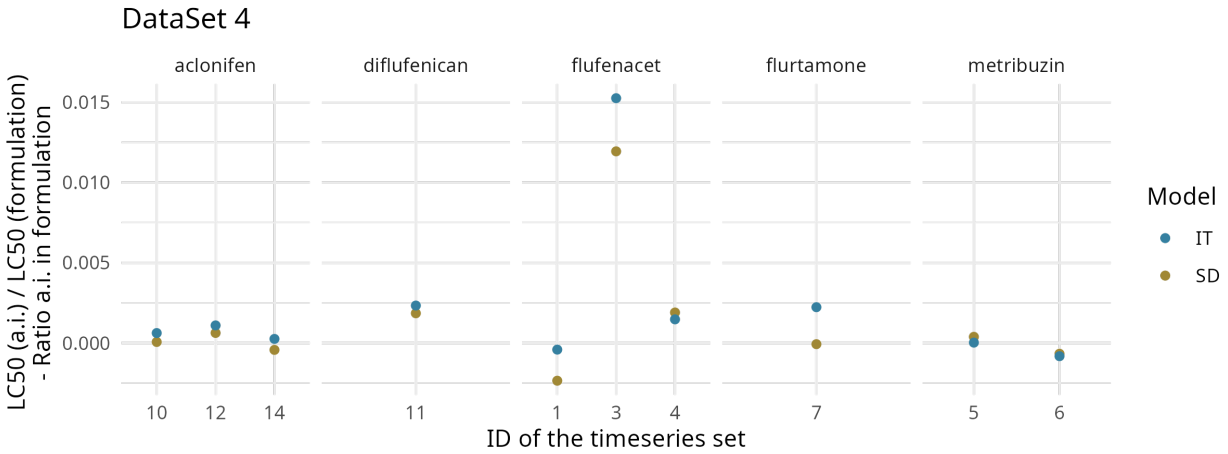
DataSet 4
aclonifen
diflufenican
flufenacet
flurtamone
metribuzin
LC50 (a.i.) / LC50 (formulation) - Ratio a.i. in formulation
0.015
0.010
0.005
0.000
10
12
14
11
1
3
4
7
5
6
ID of the timeseries set
Model
IT
SD

4 Goodness of Fit of Cross-Validation

To assess the quality of the calibration output, we used the model performance criteria recommended by EFSA in its Scientific Opinion on TKTD modeling efsa2018tktd, including the Posterior Prediction Check (PPC) and the the Normalized Root Mean Square Error (NRMSE).

The PPC enables a comparison between the predicted and observed survival counts, summarizing the predictions by their mean and the 95% uncertainty range. The percentage of predictions that capture the observed data serves as a quantitative goodness-of-fit criterion.

The NRMSE is defined by the equation:

$$\text{NRMSE} = \frac{1}{\frac{1}{N}\sum_i y_{obs,i}} \sqrt{\frac{1}{N}\sum_i \left(y_{obs,i} - y_{pred,i}\right)^2} \tag{1}$$

where $y_{obs,i}$ and $y_{pred,i}$, $i = 1, N$, are the observed and predicted values, respectively, across N data points.

Since Bayesian inference was applied, we included the Widely Applicable Information Criterion (WAIC) to estimate the effective number of parameters, accounting for overfitting watanabe2013widely. The WAIC is defined as:

$$WAIC = -2\,(\text{lpd} - \text{pWAIC}) = -2\left(\sum_i \log(E(p(y|\theta))) - \sum_i \text{var}(\log(p(y|\theta))\right) \tag{2}$$

where lpd is the log-point-wise predictive density and pWAIC represents the effective number of parameters.

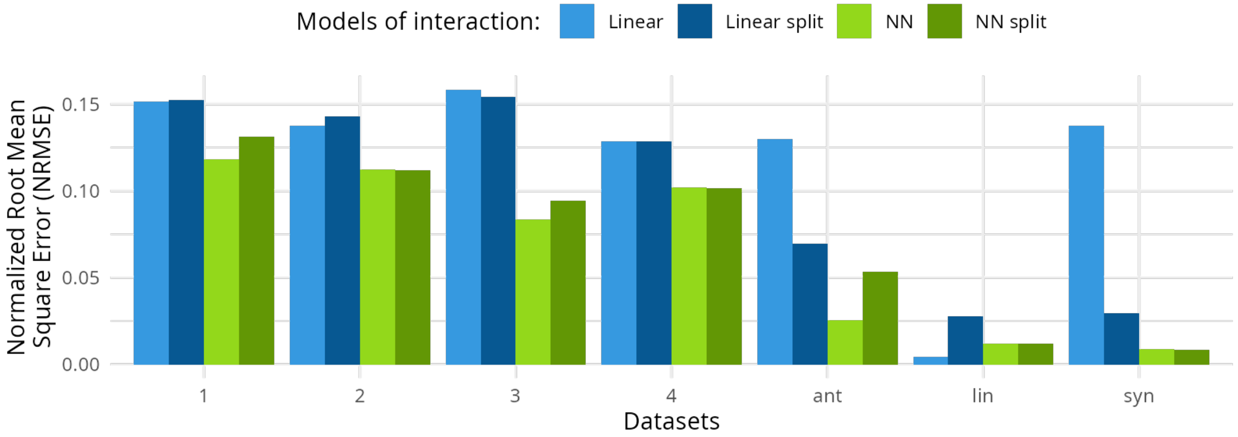

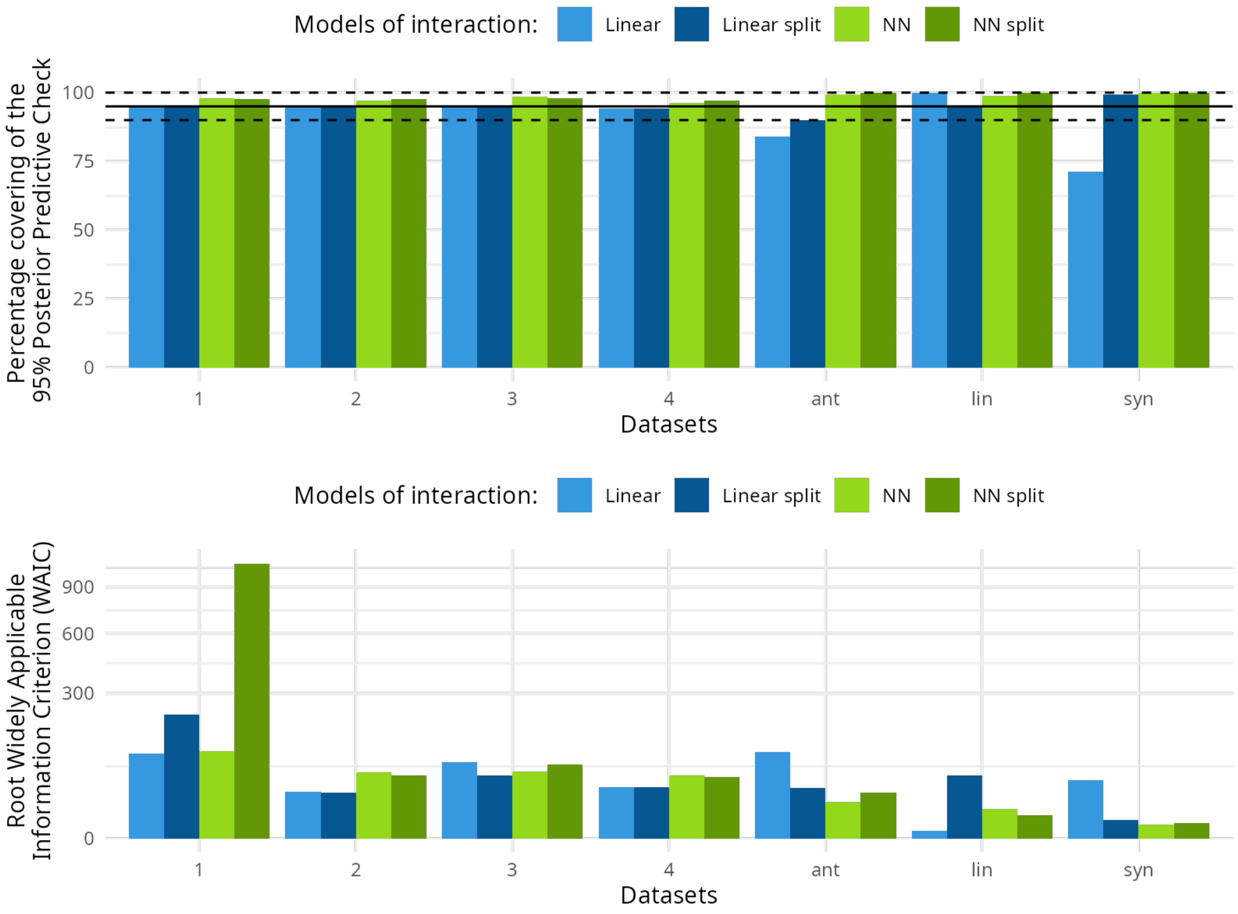
Models of interaction:
Linear
Linear split
NN
NN split
Percentage covering of the 95% Posterior Predictive Check
100
75
50
25
0
1
2
3
4
ant
lin
syn
Datasets
Models of interaction:
Linear
Linear split
NN
NN split
Root Widely Applicable Information Criterion (WAIC)
900
600
300
0
1
2
3
4
ant
lin
syn
Datasets

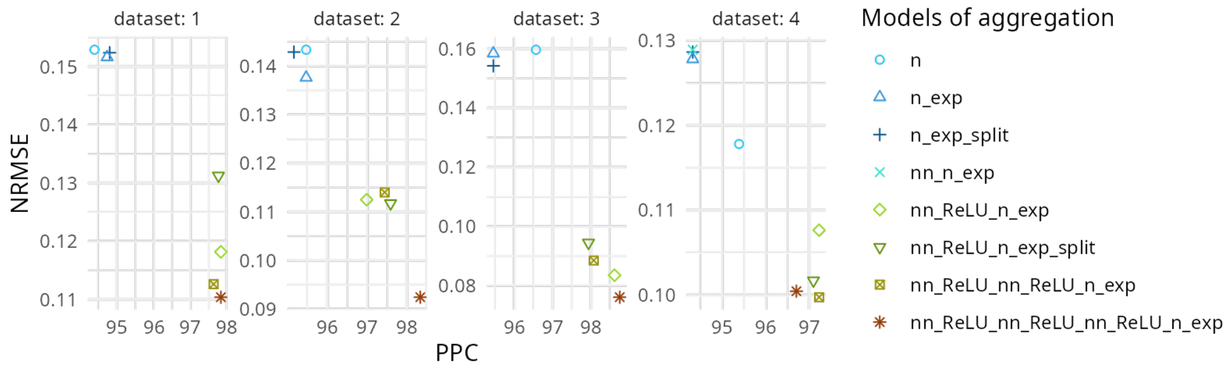

Figure D: Scatter plots illustrate the quality of calibration results for three artificial data sets fitted to five generic models (upper panel) and for four experimental data sets fitted to eight different models (lower panel). The covering of data by the Posterior Predictive Check (PPC) (x-axis) and NRMSE (y-axis) serve as goodness-of-fit metrics, with lower values indicating higher fit quality. In the "Models of aggregations" legend, `n` stands for the simplest linear model with n compounds ($b+\sum_{i=1}^{n} a_i C_i$); `n_exp` for the linear model with an exponential transformation ($\exp(b+\sum_{i=1}^{n} a_i C_i)$); `n_exp_split` is the `n_exp` model with the splitting of α; `nn_n_exp` is the application of a perceptron matrix of size $n \times n$ without activation function before the `n_exp` model; and `nn_ReLU_n_exp` is the application of an $n \times n$ layer with a `ReLU` function before the `n_exp` model. The remaining models with the `nn_ReLU` prefix involve a sequence of perceptron layers before the `n_exp` model. See Manuscript for details. The data sets called "add", "ant" and "syn" were simulated under additive, antagonism and synergism interaction hypotheses, respectively.

5 Predictions

Figure E: Results of predictions.

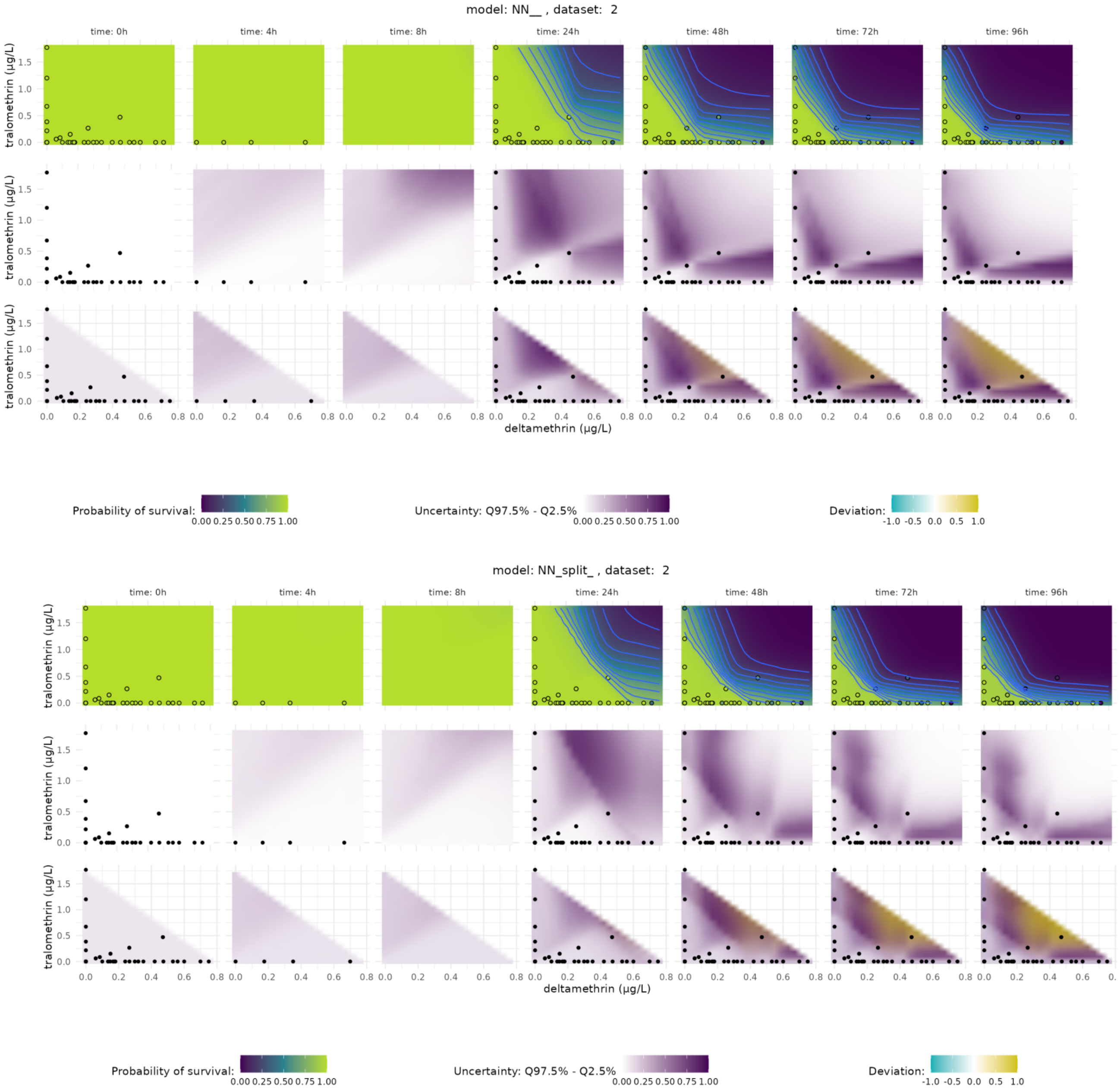

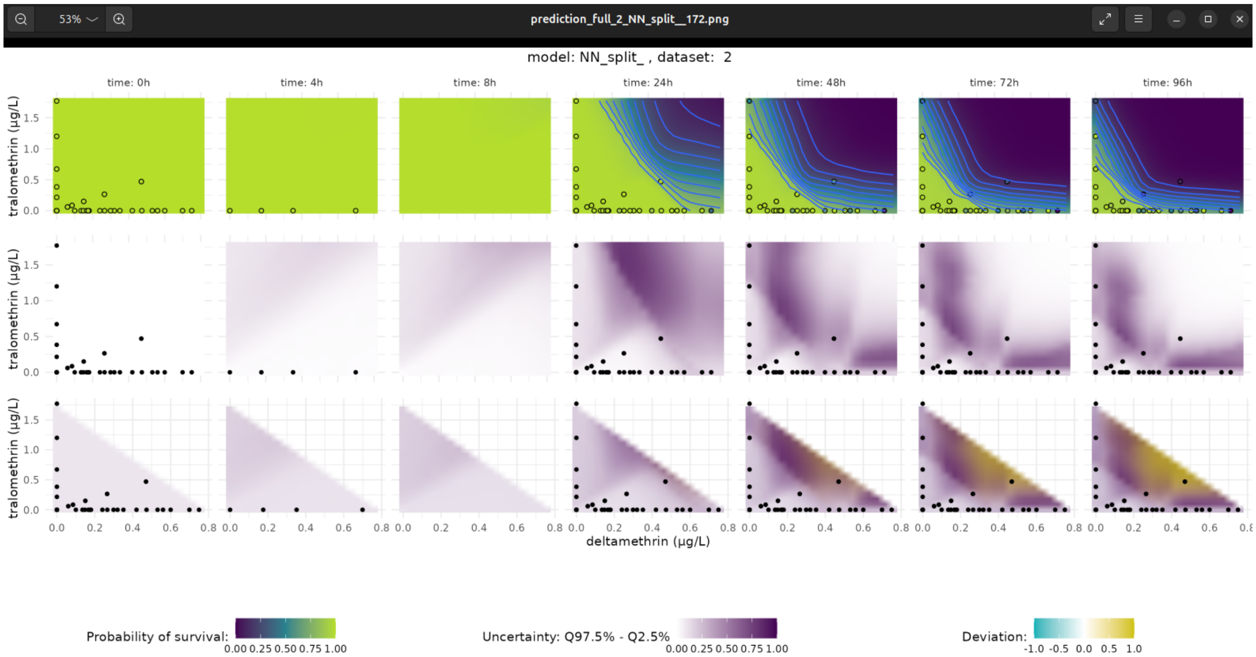
53%
prediction_full_2_NN_split__172.png
model: NN_split_ , dataset: 2
time: 0h
time: 4h
time: 8h
time: 24h
time: 48h
time: 72h
time: 96h
tralomethrin (µg/L)
deltamethrin (µg/L)
Probability of survival:
0.00 0.25 0.50 0.75 1.00
Uncertainty: Q97.5% - Q2.5%
0.00 0.25 0.50 0.75 1.00
Deviation:
-1.0 -0.5 0.0 0.5 1.0

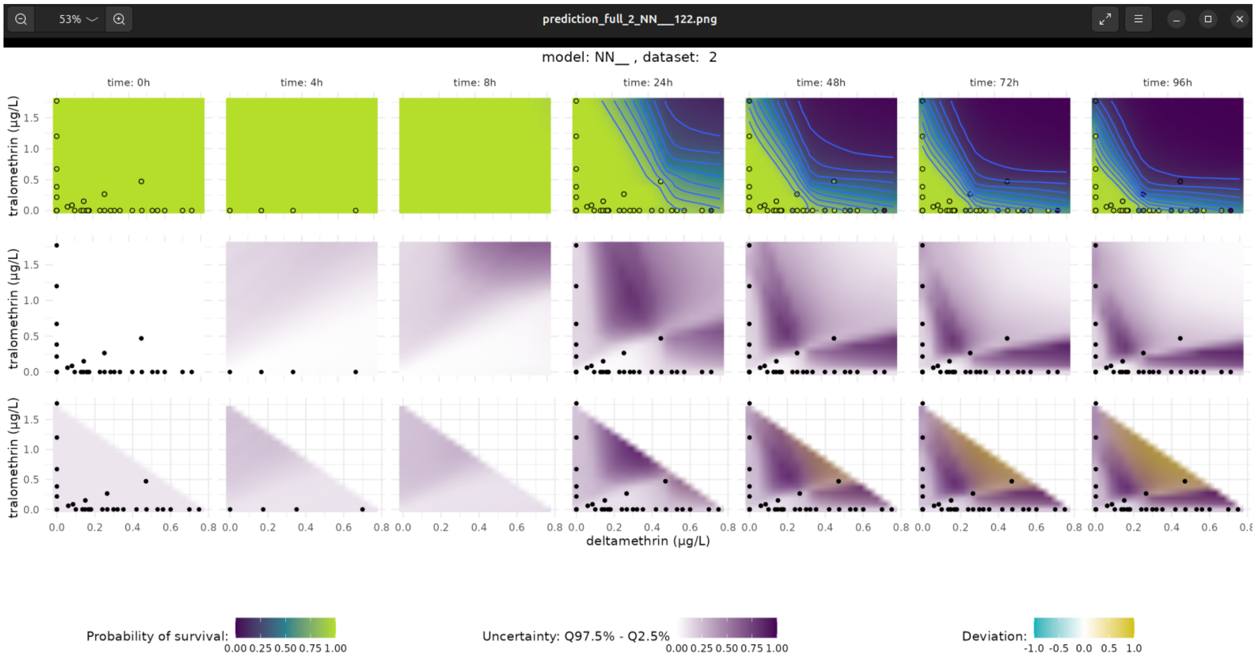
53%
prediction_full_2_NN___122.png
model: NN__ , dataset: 2
time: 0h
time: 4h
time: 8h
time: 24h
time: 48h
time: 72h
time: 96h
tralomethrin (µg/L)
deltamethrin (µg/L)
Probability of survival:
0.00 0.25 0.50 0.75 1.00
Uncertainty: Q97.5% - Q2.5%
0.00 0.25 0.50 0.75 1.00
Deviation:
-1.0 -0.5 0.0 0.5 1.0

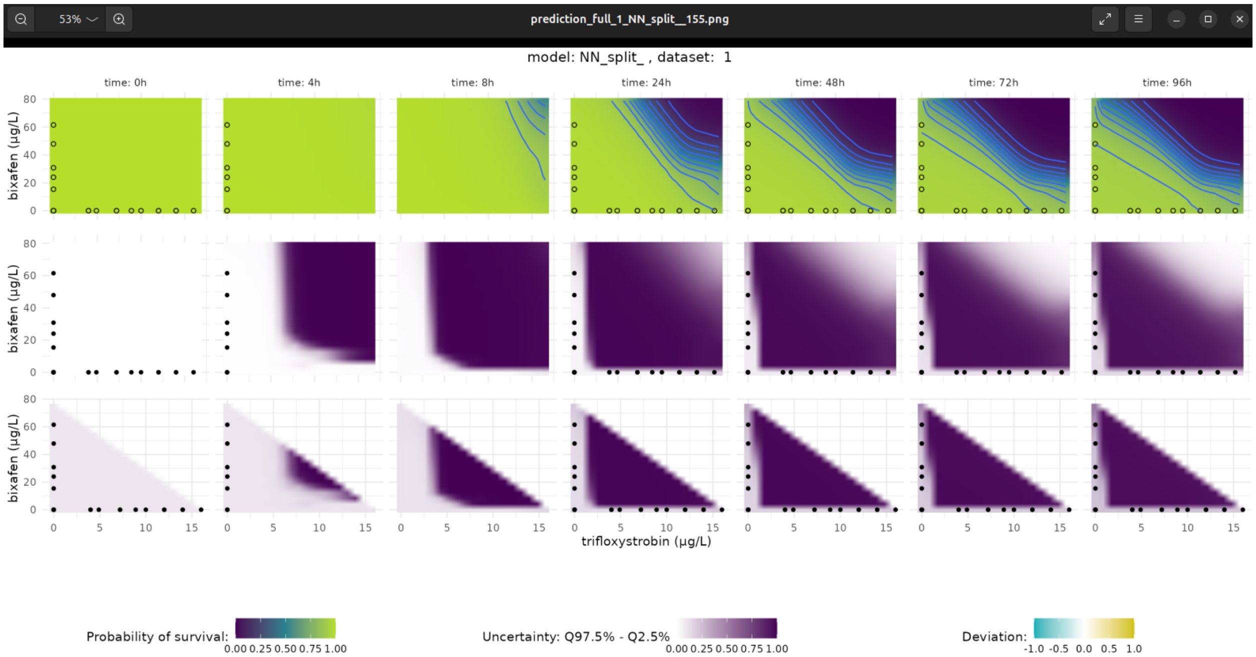
prediction_full_1_NN_split__155.png
model: NN_split_ , dataset: 1
time: 0h
time: 4h
time: 8h
time: 24h
time: 48h
time: 72h
time: 96h
bixafen (µg/L)
trifloxystrobin (µg/L)
Probability of survival:
0.00 0.25 0.50 0.75 1.00
Uncertainty: Q97.5% - Q2.5%
0.00 0.25 0.50 0.75 1.00
Deviation:
-1.0 -0.5 0.0 0.5 1.0

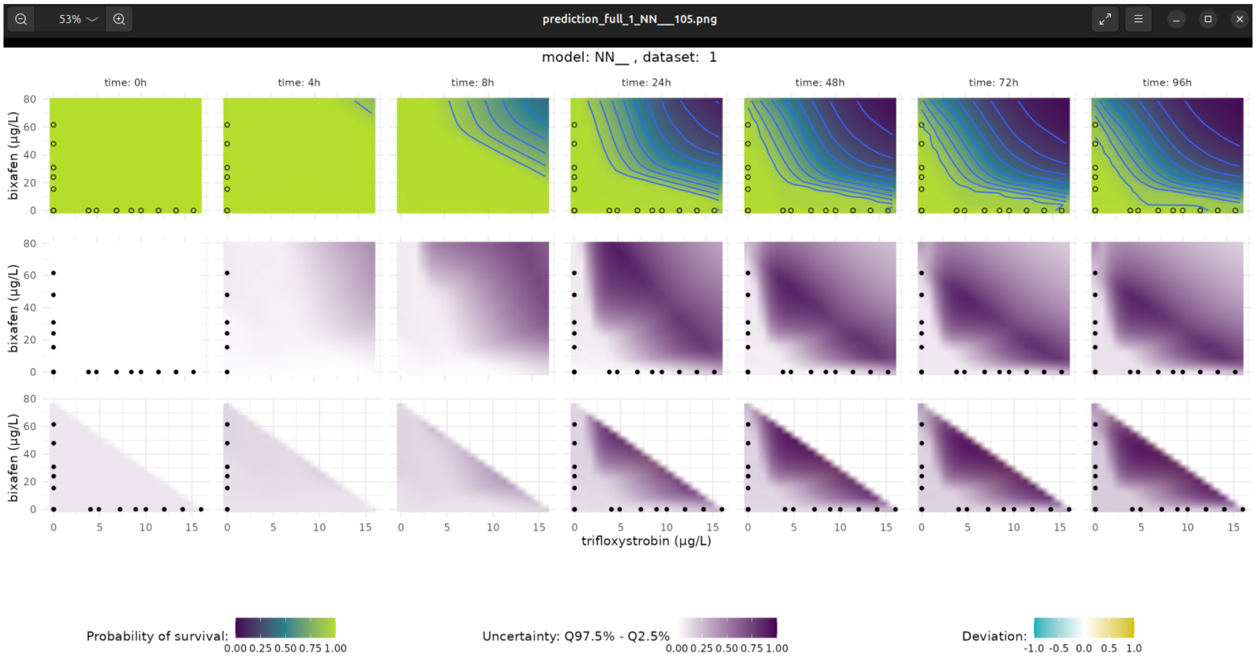
prediction_full_1_NN___105.png
model: NN__ , dataset: 1
time: 0h
time: 4h
time: 8h
time: 24h
time: 48h
time: 72h
time: 96h
bixafen (µg/L)
trifloxystrobin (µg/L)
Probability of survival:
0.00 0.25 0.50 0.75 1.00
Uncertainty: Q97.5% - Q2.5%
0.00 0.25 0.50 0.75 1.00
Deviation:
-1.0 -0.5 0.0 0.5 1.0

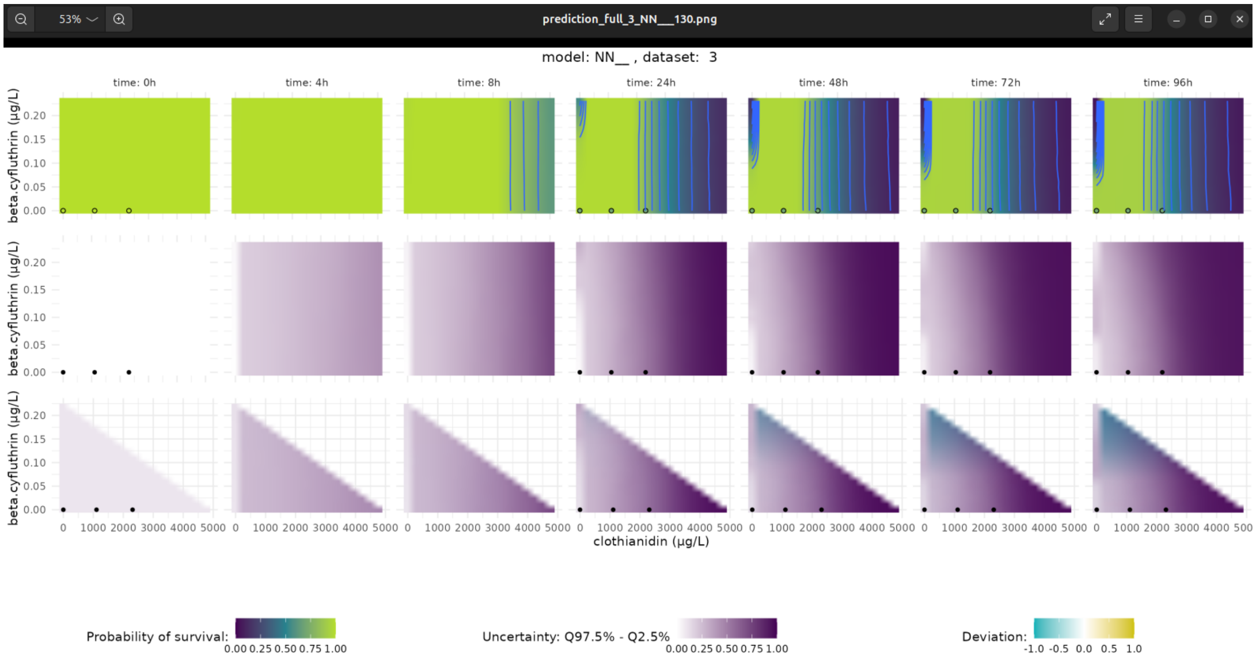
prediction_full_3_NN__130.png
model: NN__ , dataset: 3
time: 0h
time: 4h
time: 8h
time: 24h
time: 48h
time: 72h
time: 96h
beta.cyfluthrin (µg/L)
clothianidin (µg/L)
Probability of survival:
0.00 0.25 0.50 0.75 1.00
Uncertainty: Q97.5% - Q2.5%
0.00 0.25 0.50 0.75 1.00
Deviation:
-1.0 -0.5 0.0 0.5 1.0

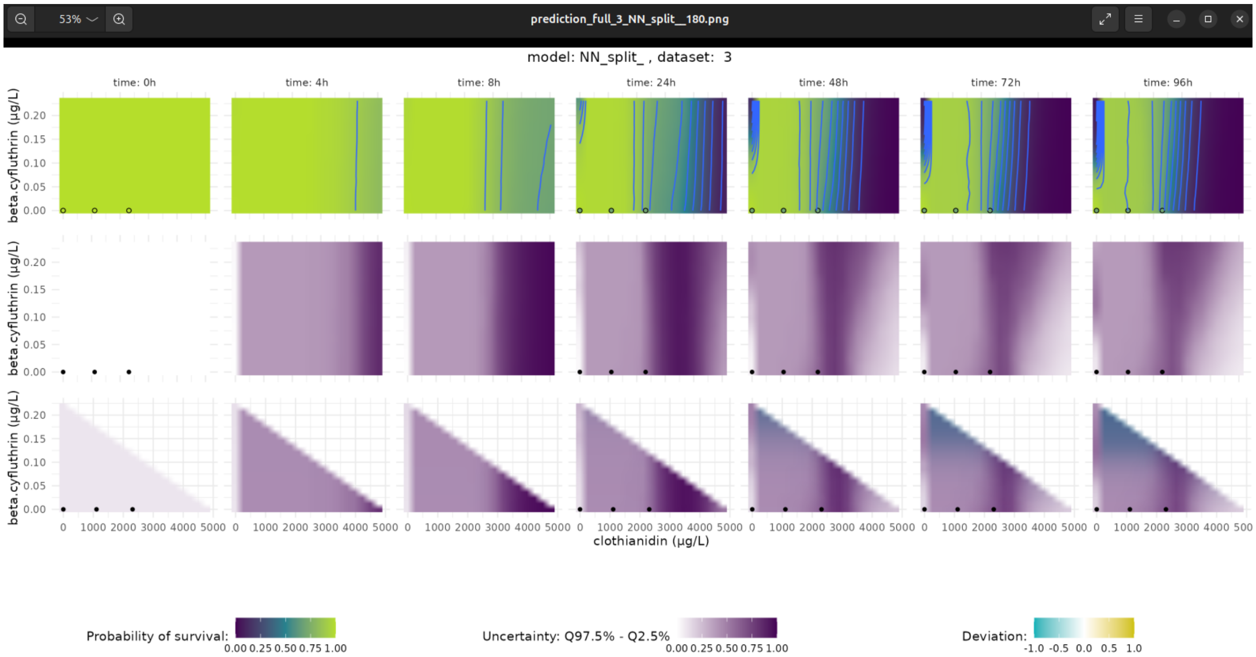
prediction_full_3_NN_split__180.png
model: NN_split_ , dataset: 3
time: 0h
time: 4h
time: 8h
time: 24h
time: 48h
time: 72h
time: 96h
beta.cyfluthrin (µg/L)
clothianidin (µg/L)
Probability of survival:
0.00 0.25 0.50 0.75 1.00
Uncertainty: Q97.5% - Q2.5%
0.00 0.25 0.50 0.75 1.00
Deviation:
-1.0 -0.5 0.0 0.5 1.0

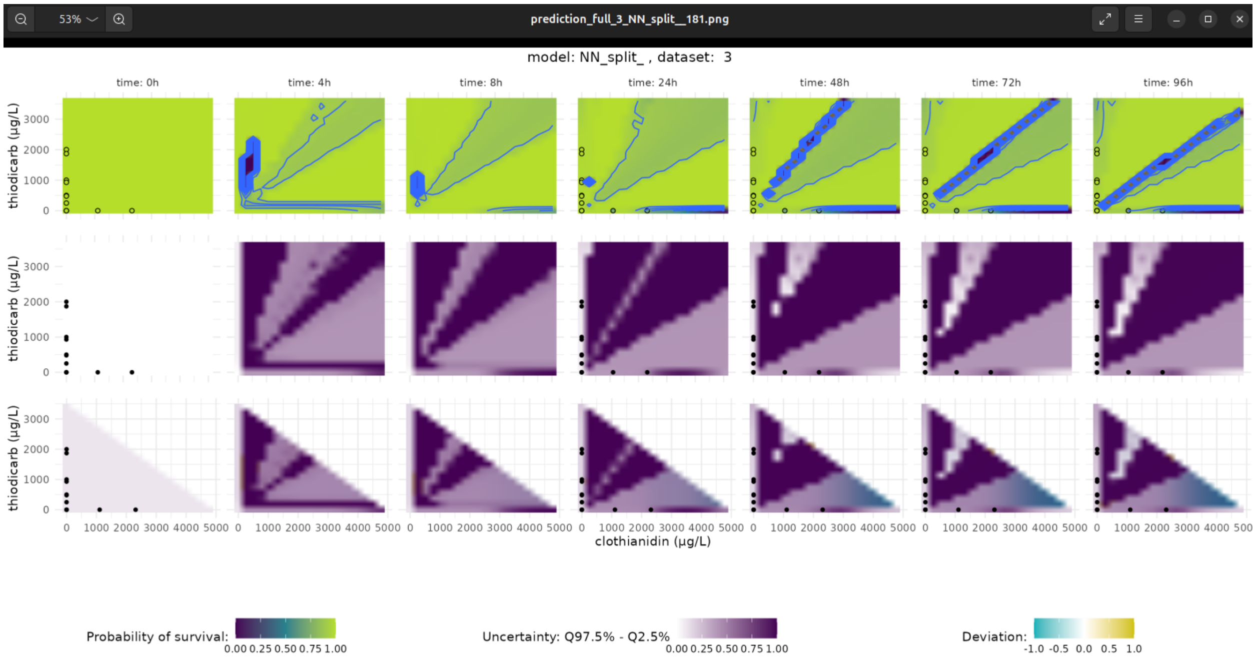
53%
prediction_full_3_NN_split__181.png
model: NN_split_ , dataset: 3
time: 0h
time: 4h
time: 8h
time: 24h
time: 48h
time: 72h
time: 96h
thiodicarb (µg/L)
clothianidin (µg/L)
Probability of survival:
0.00 0.25 0.50 0.75 1.00
Uncertainty: Q97.5% - Q2.5%
0.00 0.25 0.50 0.75 1.00
Deviation:
-1.0 -0.5 0.0 0.5 1.0

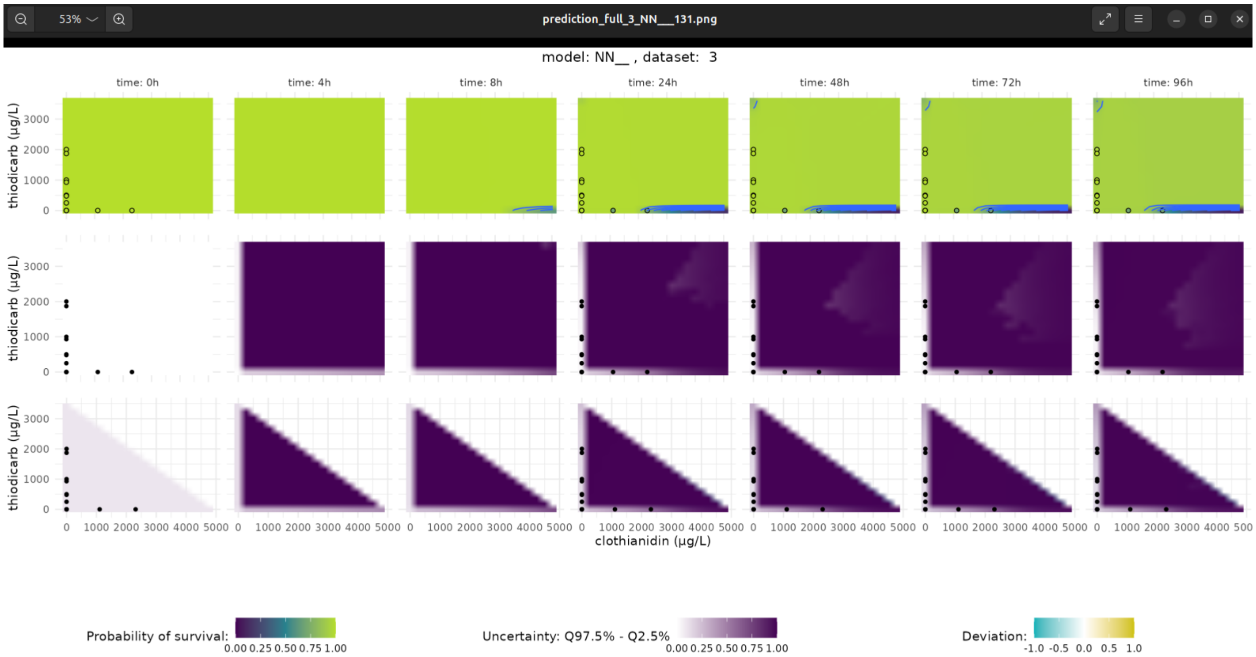
53%
prediction_full_3_NN__131.png
model: NN__ , dataset: 3
time: 0h
time: 4h
time: 8h
time: 24h
time: 48h
time: 72h
time: 96h
thiodicarb (µg/L)
clothianidin (µg/L)
Probability of survival:
0.00 0.25 0.50 0.75 1.00
Uncertainty: Q97.5% - Q2.5%
0.00 0.25 0.50 0.75 1.00
Deviation:
-1.0 -0.5 0.0 0.5 1.0

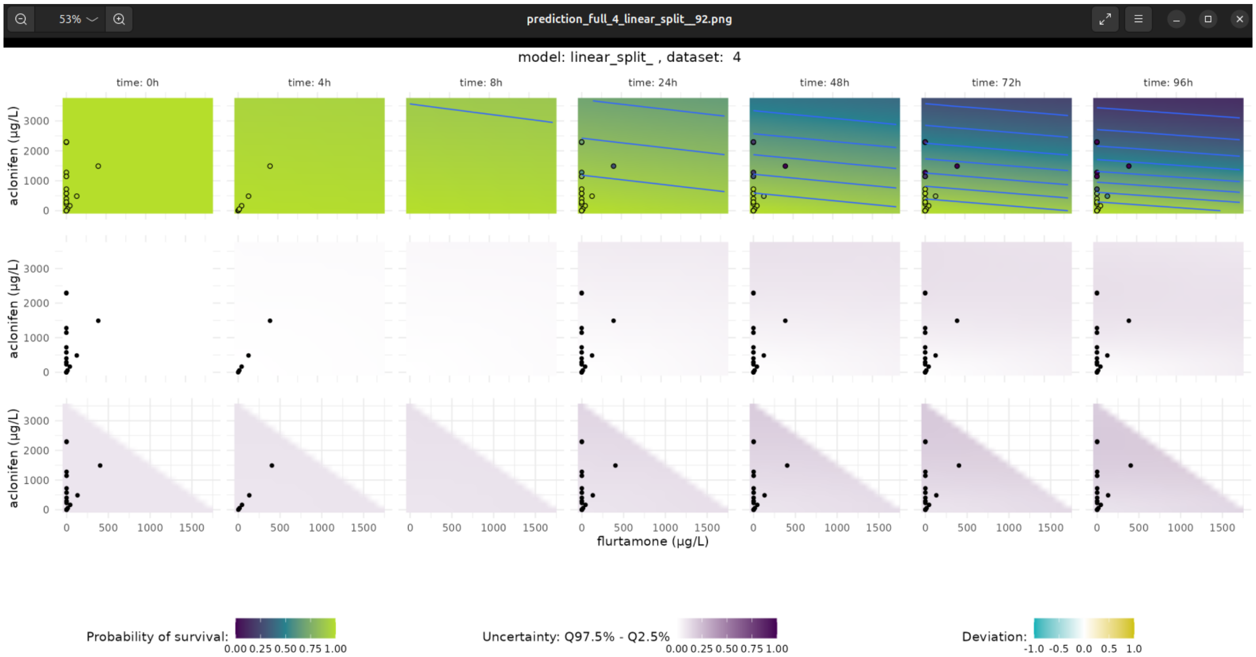
prediction_full_4_linear_split__92.png
model: linear_split_ , dataset: 4
time: 0h
time: 4h
time: 8h
time: 24h
time: 48h
time: 72h
time: 96h
aclonifen (µg/L)
flurtamone (µg/L)
Probability of survival:
0.00 0.25 0.50 0.75 1.00
Uncertainty: Q97.5% - Q2.5%
0.00 0.25 0.50 0.75 1.00
Deviation:
-1.0 -0.5 0.0 0.5 1.0

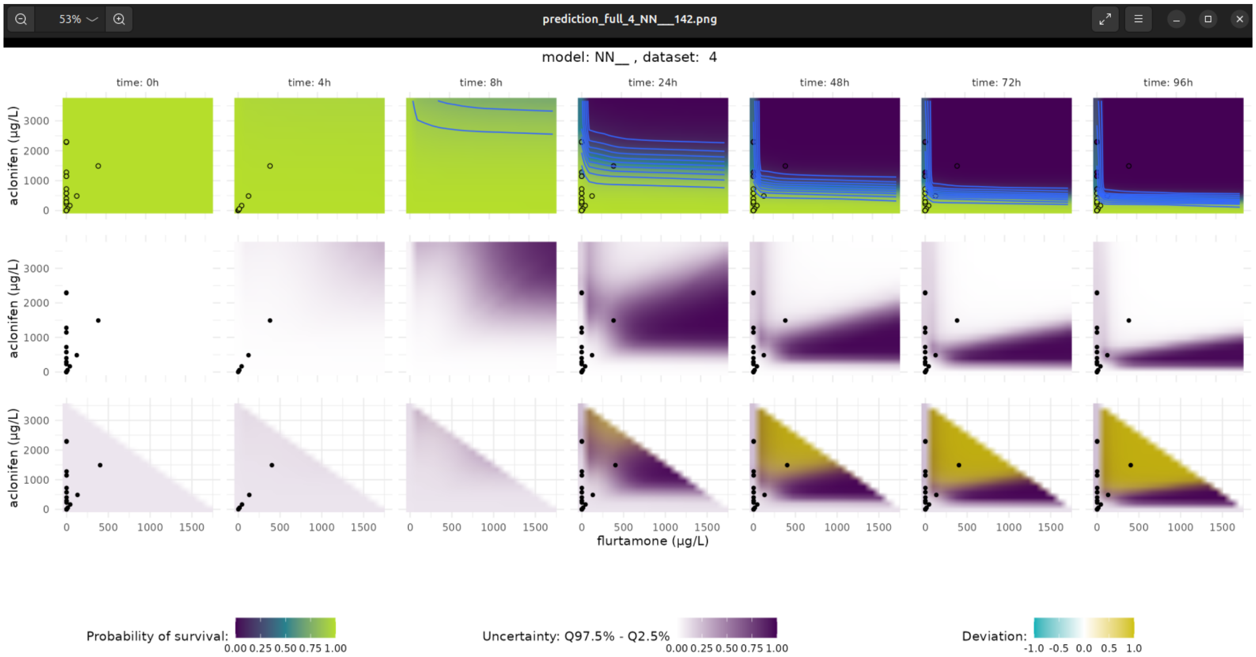
prediction_full_4_NN__142.png
model: NN__ , dataset: 4
time: 0h
time: 4h
time: 8h
time: 24h
time: 48h
time: 72h
time: 96h
aclonifen (µg/L)
flurtamone (µg/L)
Probability of survival:
0.00 0.25 0.50 0.75 1.00
Uncertainty: Q97.5% - Q2.5%
0.00 0.25 0.50 0.75 1.00
Deviation:
-1.0 -0.5 0.0 0.5 1.0

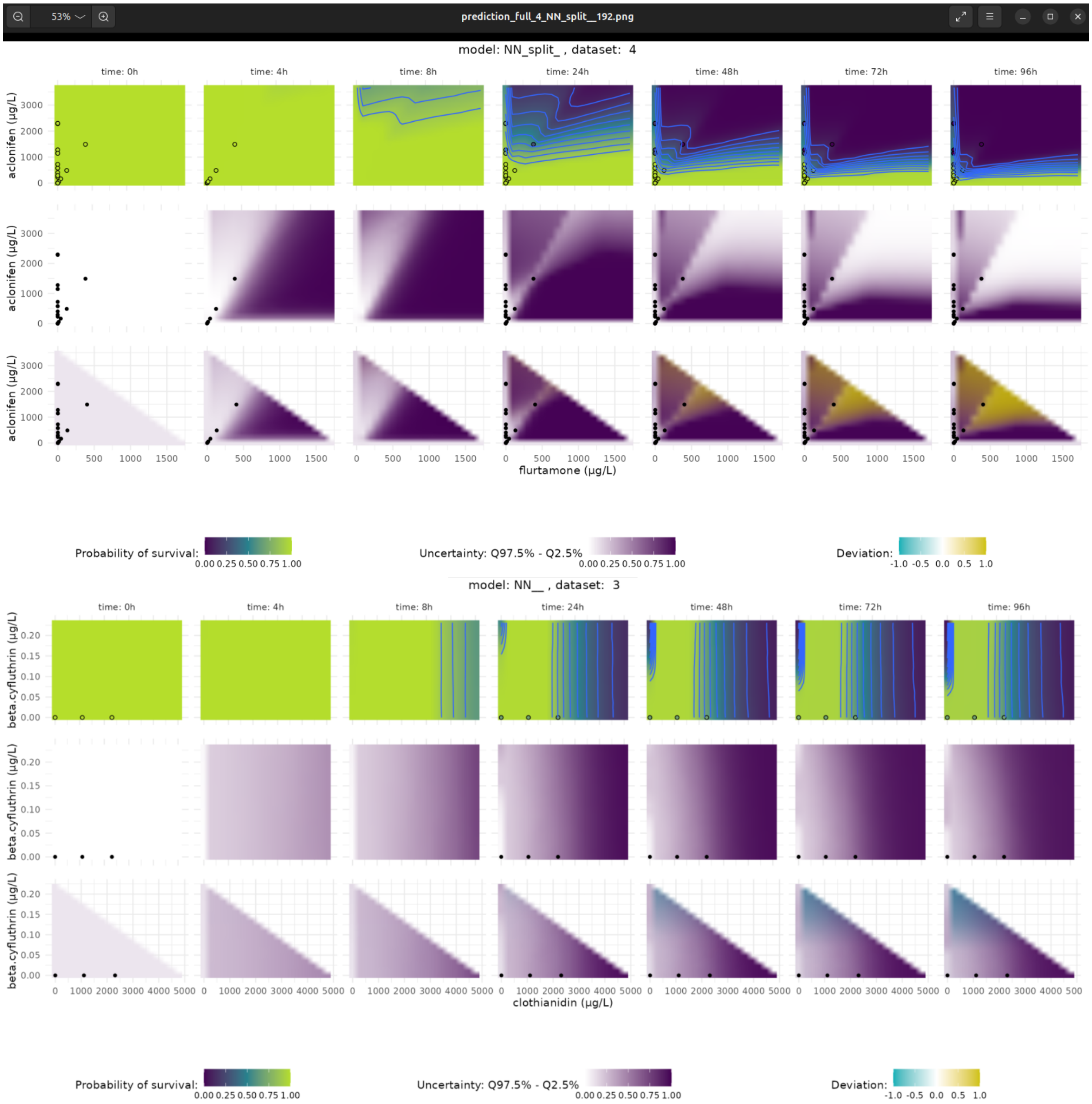
prediction_full_4_NN_split__192.png
53%
model: NN_split_ , dataset: 4
time: 0h
time: 4h
time: 8h
time: 24h
time: 48h
time: 72h
time: 96h
aclonifen (µg/L)
flurtamone (µg/L)
Probability of survival:
0.00 0.25 0.50 0.75 1.00
Uncertainty: Q97.5% - Q2.5%
0.00 0.25 0.50 0.75 1.00
Deviation:
-1.0 -0.5 0.0 0.5 1.0
model: NN__ , dataset: 3
time: 0h
time: 4h
time: 8h
time: 24h
time: 48h
time: 72h
time: 96h
beta.cyfluthrin (µg/L)
clothianidin (µg/L)
Probability of survival:
0.00 0.25 0.50 0.75 1.00
Uncertainty: Q97.5% - Q2.5%
0.00 0.25 0.50 0.75 1.00
Deviation:
-1.0 -0.5 0.0 0.5 1.0

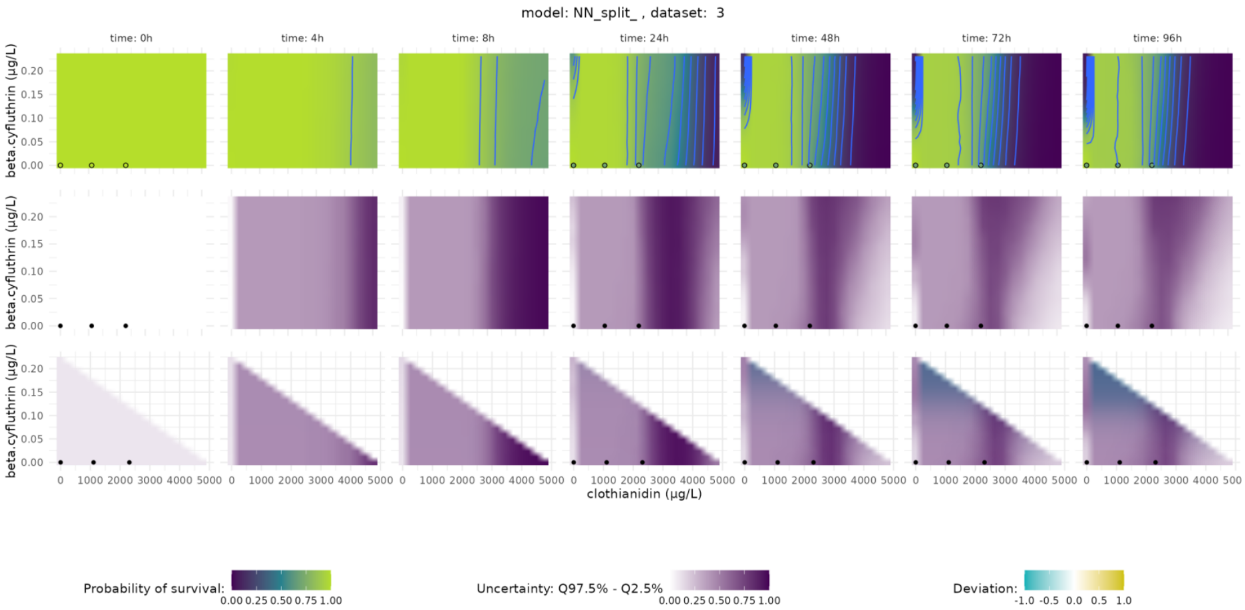
model: NN_split_ , dataset: 3
time: 0h
time: 4h
time: 8h
time: 24h
time: 48h
time: 72h
time: 96h
beta.cyfluthrin (µg/L)
0.00 0.05 0.10 0.15 0.20
0 1000 2000 3000 4000 5000
clothianidin (µg/L)
Probability of survival:
0.00 0.25 0.50 0.75 1.00
Uncertainty: Q97.5% - Q2.5%
0.00 0.25 0.50 0.75 1.00
Deviation:
-1.0 -0.5 0.0 0.5 1.0

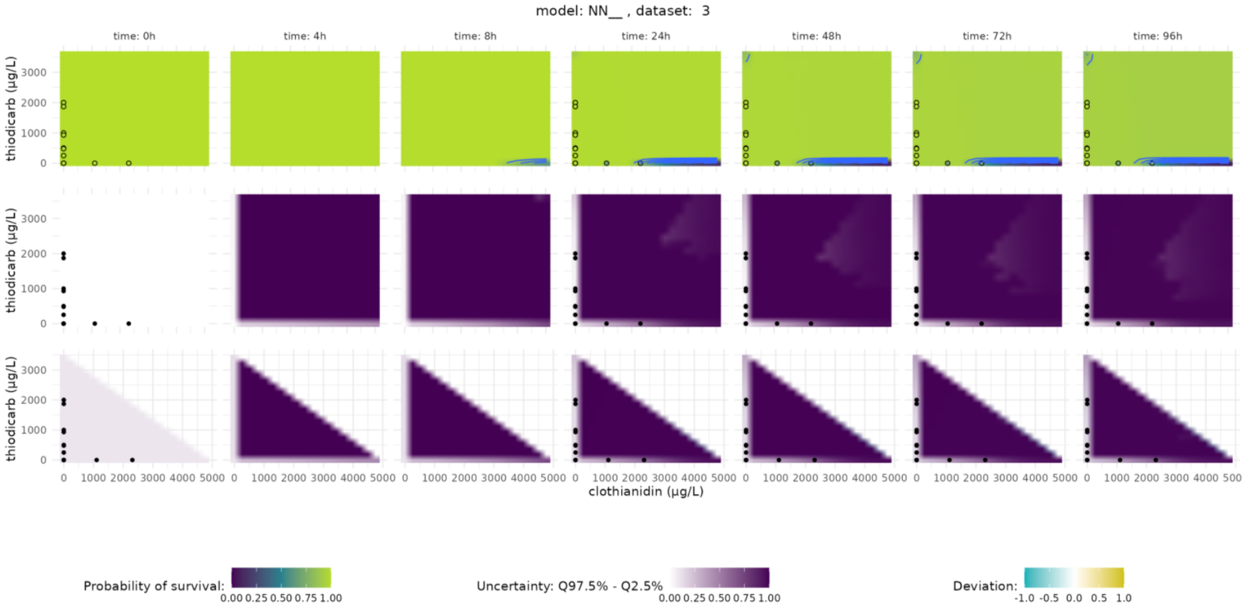
model: NN__ , dataset: 3
time: 0h
time: 4h
time: 8h
time: 24h
time: 48h
time: 72h
time: 96h
thiodicarb (µg/L)
0 1000 2000 3000
0 1000 2000 3000 4000 5000
clothianidin (µg/L)
Probability of survival:
0.00 0.25 0.50 0.75 1.00
Uncertainty: Q97.5% - Q2.5%
0.00 0.25 0.50 0.75 1.00
Deviation:
-1.0 -0.5 0.0 0.5 1.0

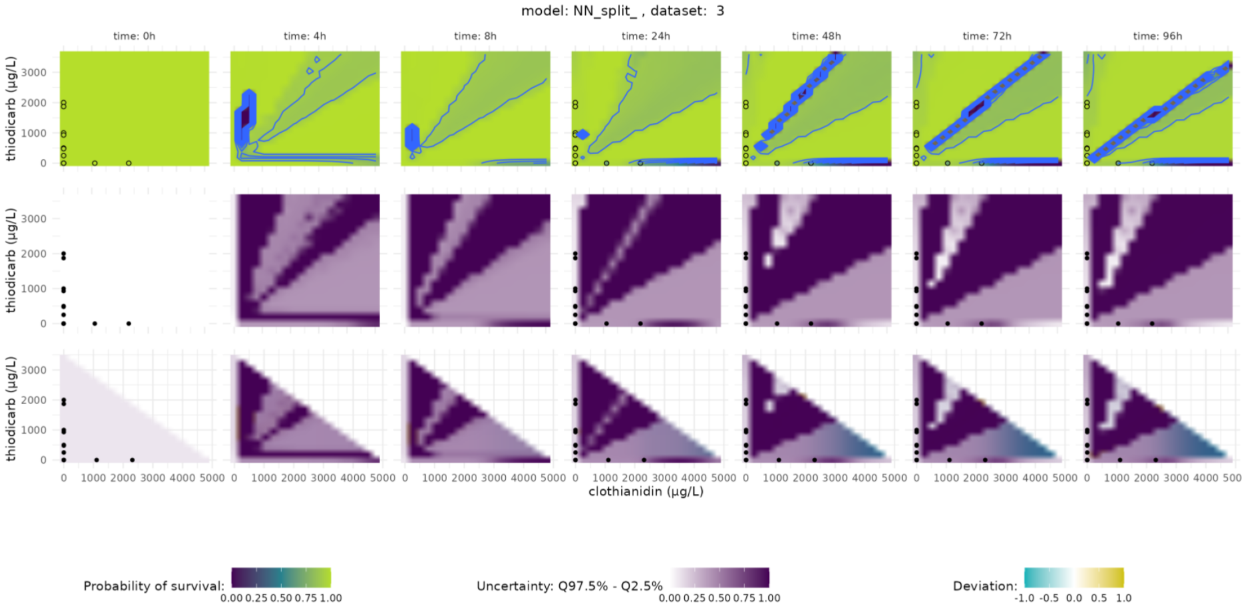
model: NN_split_ , dataset: 3
time: 0h
time: 4h
time: 8h
time: 24h
time: 48h
time: 72h
time: 96h
thiodicarb (µg/L)
clothianidin (µg/L)
Probability of survival:
0.00 0.25 0.50 0.75 1.00
Uncertainty: Q97.5% - Q2.5%
0.00 0.25 0.50 0.75 1.00
Deviation:
-1.0 -0.5 0.0 0.5 1.0

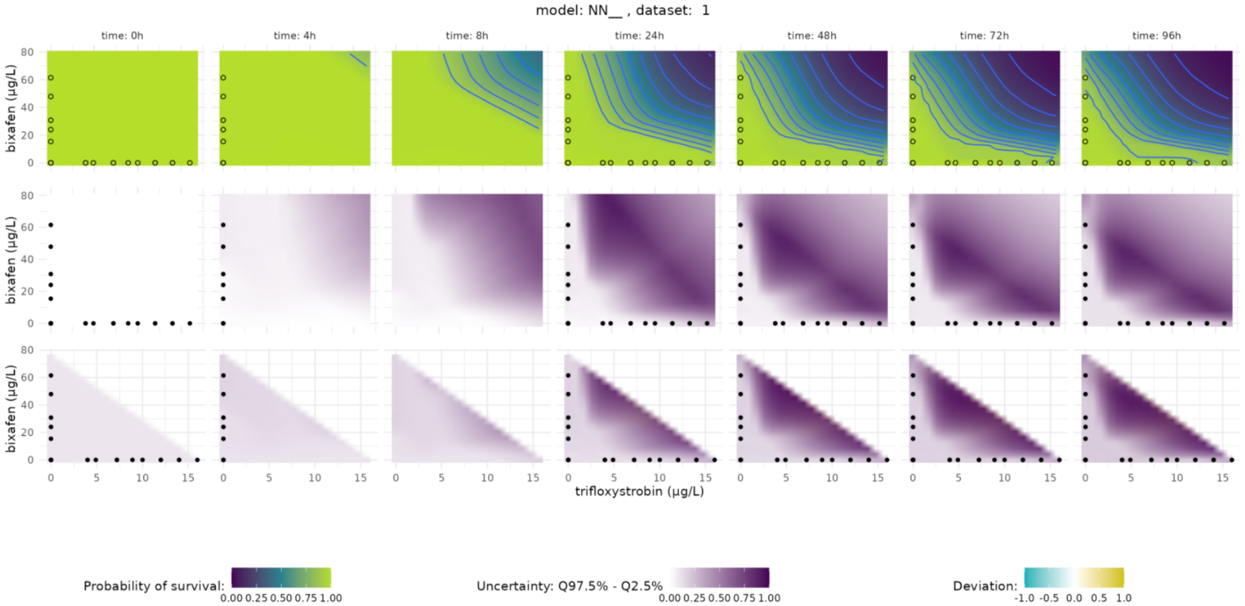
model: NN__ , dataset: 1
time: 0h
time: 4h
time: 8h
time: 24h
time: 48h
time: 72h
time: 96h
bixafen (µg/L)
trifloxystrobin (µg/L)
Probability of survival:
0.00 0.25 0.50 0.75 1.00
Uncertainty: Q97.5% - Q2.5%
0.00 0.25 0.50 0.75 1.00
Deviation:
-1.0 -0.5 0.0 0.5 1.0

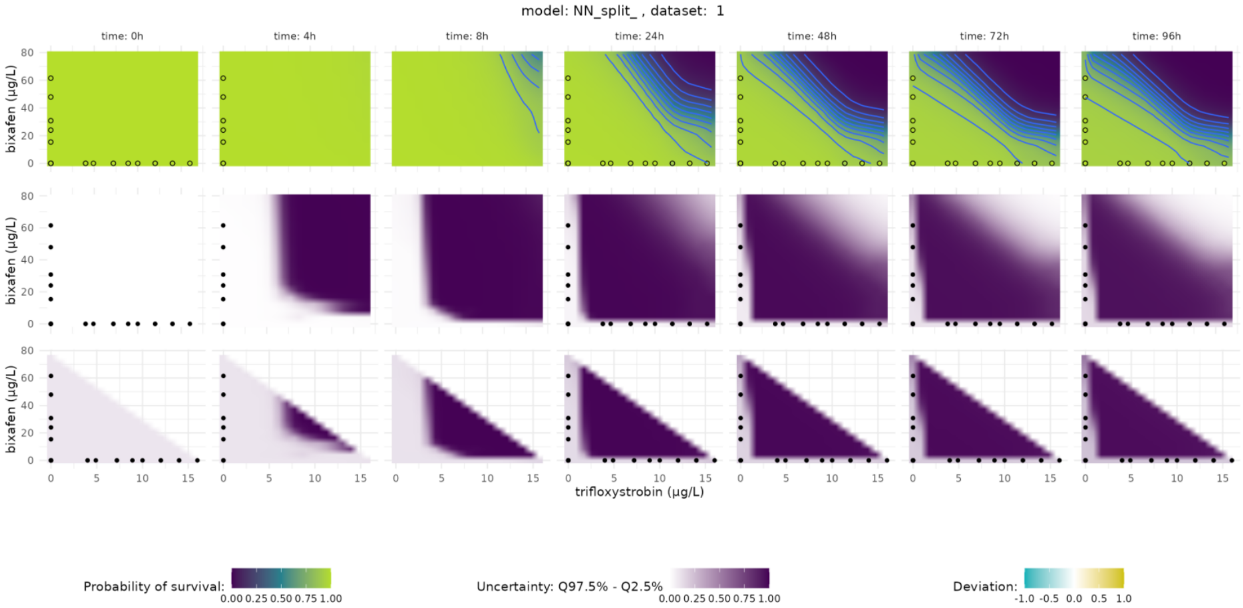
model: NN_split_ , dataset: 1
time: 0h
time: 4h
time: 8h
time: 24h
time: 48h
time: 72h
time: 96h
bixafen (µg/L)
trifloxystrobin (µg/L)
Probability of survival:
0.00 0.25 0.50 0.75 1.00
Uncertainty: Q97.5% - Q2.5%
0.00 0.25 0.50 0.75 1.00
Deviation:
-1.0 -0.5 0.0 0.5 1.0

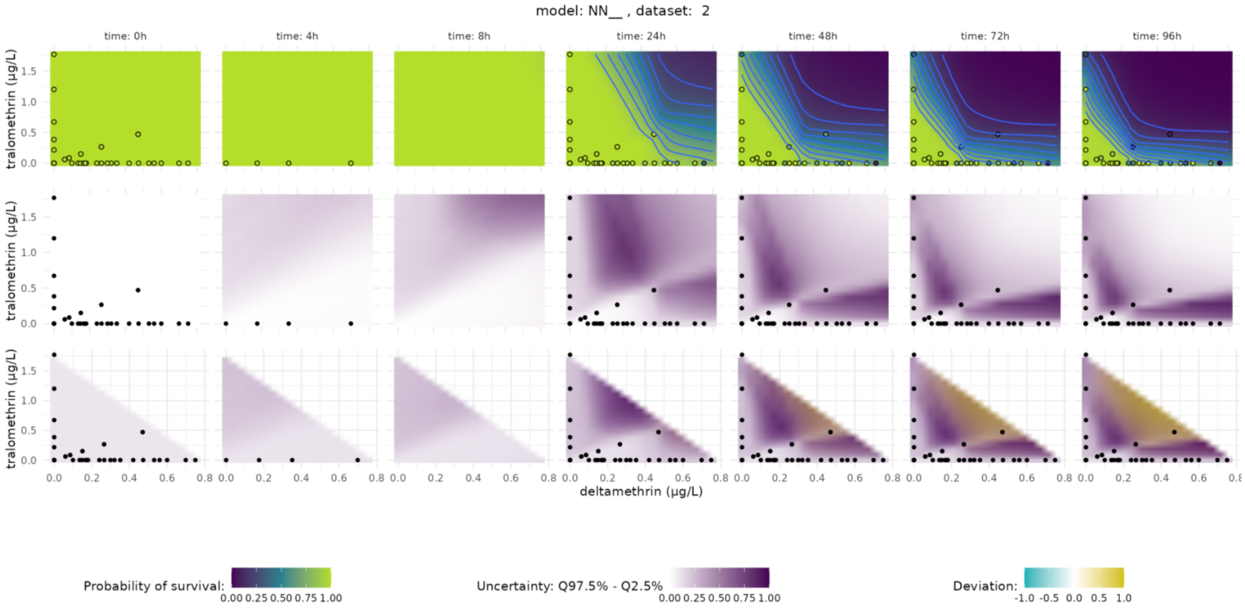
model: NN__ , dataset: 2
time: 0h
time: 4h
time: 8h
time: 24h
time: 48h
time: 72h
time: 96h
tralomethrin (µg/L)
deltamethrin (µg/L)
Probability of survival:
0.00 0.25 0.50 0.75 1.00
Uncertainty: Q97.5% - Q2.5%
0.00 0.25 0.50 0.75 1.00
Deviation:
-1.0 -0.5 0.0 0.5 1.0

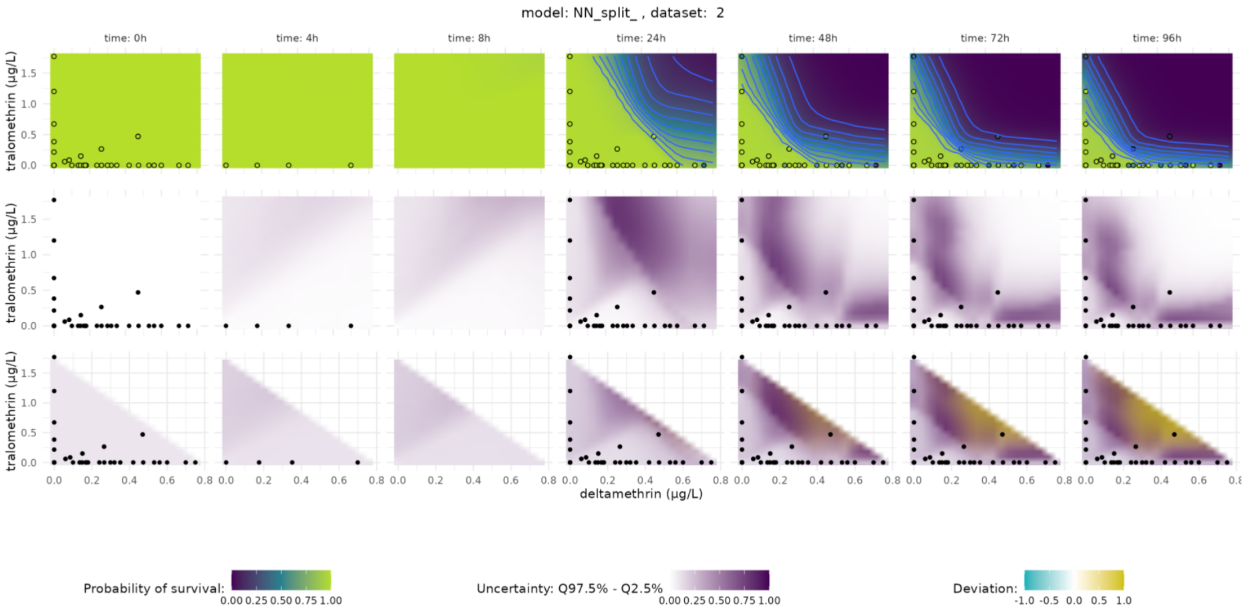
model: NN_split_ , dataset: 2
time: 0h
time: 4h
time: 8h
time: 24h
time: 48h
time: 72h
time: 96h
tralomethrin (µg/L)
deltamethrin (µg/L)
Probability of survival:
0.00 0.25 0.50 0.75 1.00
Uncertainty: Q97.5% - Q2.5%
0.00 0.25 0.50 0.75 1.00
Deviation:
-1.0 -0.5 0.0 0.5 1.0

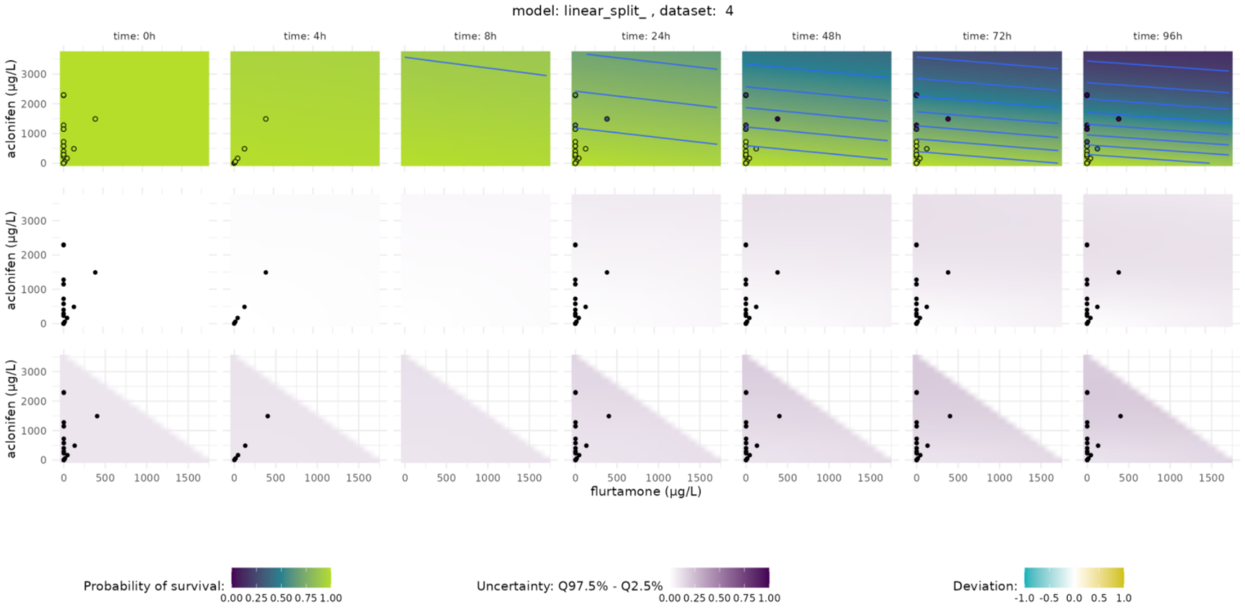
model: linear_split_ , dataset: 4
time: 0h
time: 4h
time: 8h
time: 24h
time: 48h
time: 72h
time: 96h
aclonifen (µg/L)
flurtamone (µg/L)
Probability of survival:
0.00 0.25 0.50 0.75 1.00
Uncertainty: Q97.5% - Q2.5%
0.00 0.25 0.50 0.75 1.00
Deviation:
-1.0 -0.5 0.0 0.5 1.0

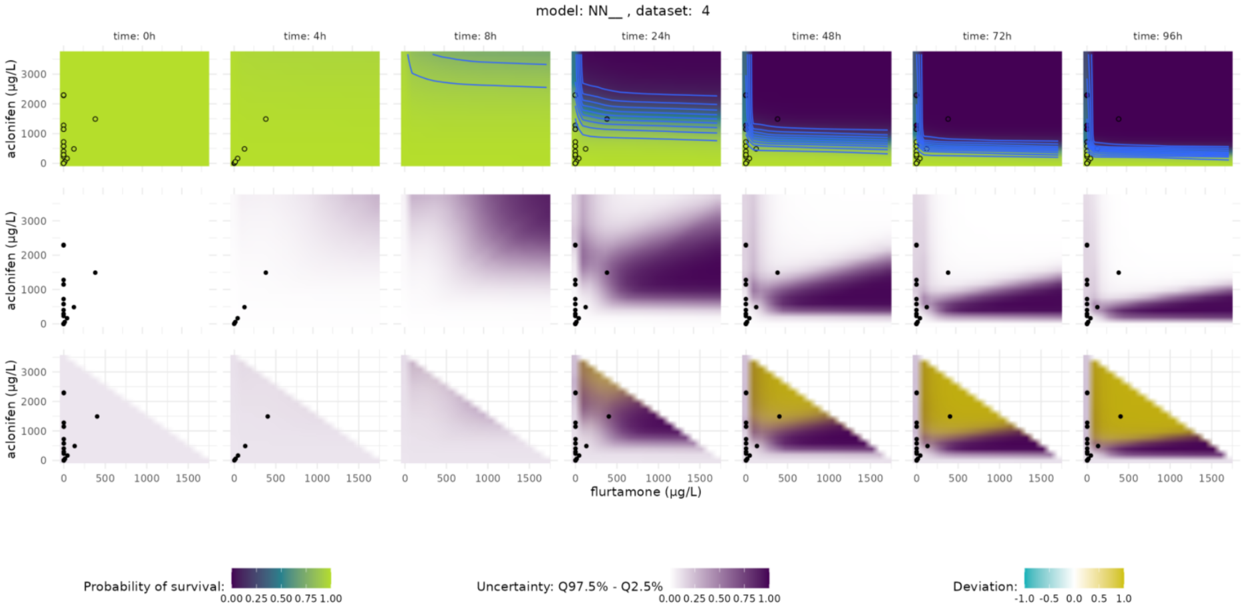
model: NN__ , dataset: 4
time: 0h
time: 4h
time: 8h
time: 24h
time: 48h
time: 72h
time: 96h
aclonifen (µg/L)
0
1000
2000
3000
0
500
1000
1500
flurtamone (µg/L)
Probability of survival:
0.00 0.25 0.50 0.75 1.00
Uncertainty: Q97.5% - Q2.5%
0.00 0.25 0.50 0.75 1.00
Deviation:
-1.0 -0.5 0.0 0.5 1.0

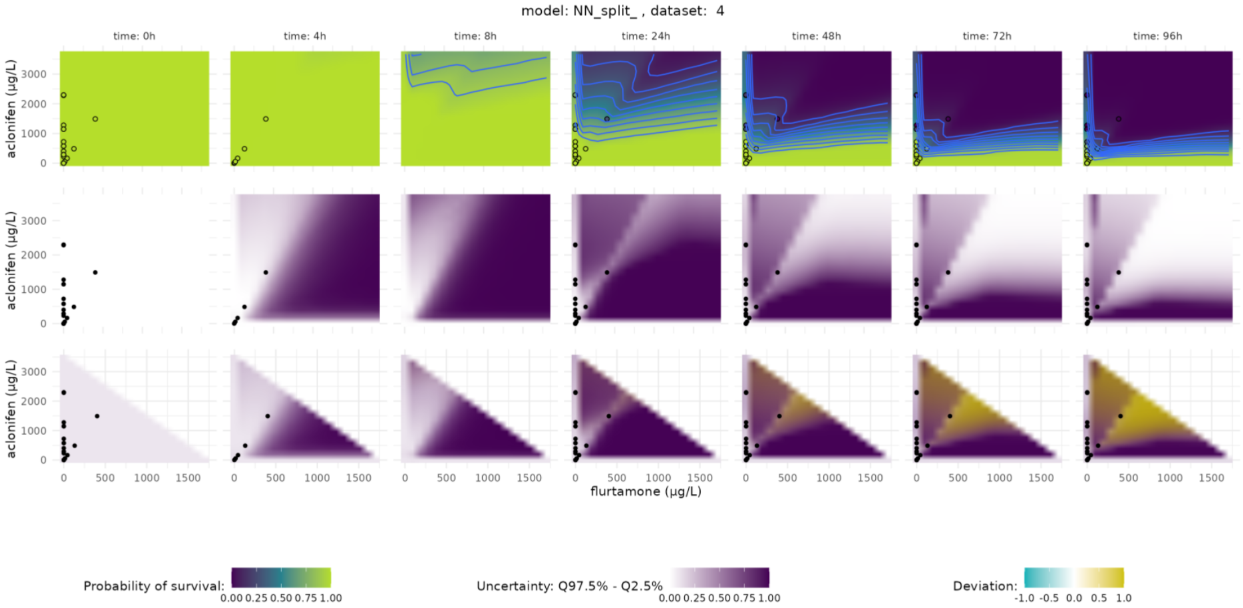
model: NN_split_ , dataset: 4
time: 0h
time: 4h
time: 8h
time: 24h
time: 48h
time: 72h
time: 96h
aclonifen (µg/L)
0
1000
2000
3000
0
500
1000
1500
flurtamone (µg/L)
Probability of survival:
0.00 0.25 0.50 0.75 1.00
Uncertainty: Q97.5% - Q2.5%
0.00 0.25 0.50 0.75 1.00
Deviation:
-1.0 -0.5 0.0 0.5 1.0
