# Supplementary figures and images for: A Bayesian neural ordinary differential equations framework to study the effects of chemical mixtures on survival
Source: PLoS Comput Biol. 2025 Nov 10;21(11):e1013681. doi: 10.1371/journal.pcbi.1013681 (PMC12674509; doi:10.1371/journal.pcbi.1013681)

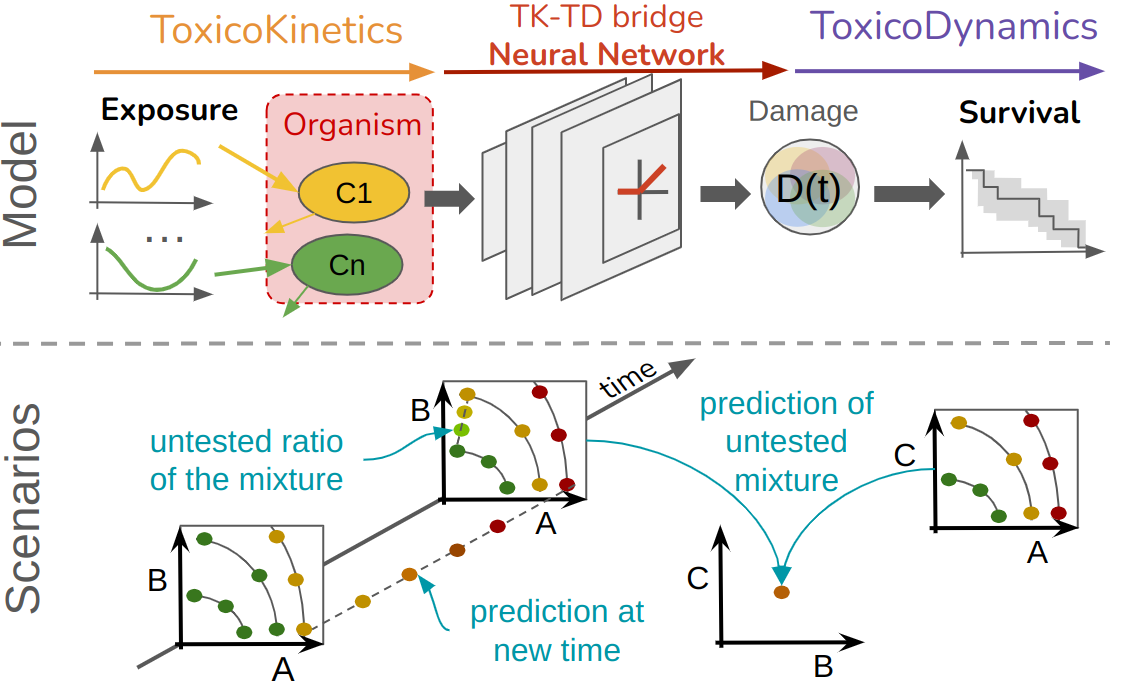

Supplement: S1 Fig — (TIFF) [file pcbi.1013681.s003.tiff]
