# Supplementary material for: A Bayesian neural ordinary differential equations framework to study the effects of chemical mixtures on survival
Source: PLoS Comput Biol. 2025 Nov 10;21(11):e1013681. doi: 10.1371/journal.pcbi.1013681 (PMC12674509; doi:10.1371/journal.pcbi.1013681)
Supplement: Attachment — Submitted filename: Response to Reviewer _ PLOS Computational Biology.pdf [file pcbi.1013681.s004.pdf]

Virgile BAUDROT
Qonfluens SAS, Montpellier
virgile.baudrot@qonfluens.com

Editor
PLOS Computational Biology

Dear Editor,

Thank you for reviewing the manuscript. We are also deeply grateful to the four reviewers for dedicating their time, expertise, and for providing such constructive feedback. Their insightful comments were invaluable in guiding the revision of our work, and we believe it is now significantly improved.
We have carefully considered and addressed every point raised by you and the reviewers. Below, we provide a point-by-point response detailing the changes made.

All comments have been carefully addressed. We are confident that these revisions have fully addressed the concerns raised, resulting in a much stronger manuscript. We look forward to receiving your acceptance of our revised work.

Sincerely,

Virgile Baudrot on behalf of all authors.

Additional Editor Comments:

I agree with the reviewers that this is an interesting study that is likely to be of relevance in the field. However, I also agree with the reviewers (especially R2), that the comparison between the neural network model and other approaches is difficult to interpret and should be strengthened during revision. Reviewer 2 provides a number of suggestions here, which I suggest the authors pay careful attention to during their revision. I want to stress the importance of using an entirely held-out validation data set for a final comparison. While cross-validation is critical for training, it can be insufficient to rely solely on cross-validation for final model comparison. Additionally, the reviewers noted that more information is needed on how the model was calibrated and how the authors determined that it had fully converged. Lastly, the reviewers raised concerns about whether all necessary data/code/etc were made available. If there are aspects of the study which cannot be released publicly, then the authors must request a waiver per the journal's policy.

Thank you for this positive assessment.

Following the comments from Reviewer #2, we have revised the manuscript with regards to the role of neural network (NN) models. The revised text no longer presents the NN as the default choice. Instead, we now present the simpler, linear-bridge model as the parsimonious and robust baseline, particularly for regulatory applications where additivity is the primary assumption. We now position the NN model as a powerful, data-intensive tool for exploratory analysis, to be employed selectively when model selection criteria or prior toxicological knowledge suggest the presence of significant non-linear interactions.

We acknowledge that the original submission lacked sufficient detail on the Bayesian calibration process. We have expanded the implementation section to provide exhaustive details on the MCMC sampler settings (chains, iterations, algorithm, convergence diagnostics), and have added all prior specifications to the manuscript (new Table 2) and in supplementary material, addressing key concerns raised by Reviewers #1, #2, and #3.

An important concern, raised by you and Reviewer #2, was the availability of data and code. Note that all raw survival data, exposure concentrations, and the complete R analysis scripts required to reproduce every result, figure, and table in the manuscript are now publicly available without restriction in a dedicated GitHub repository. This repository can be accessed at: https://github.com/virgile-baudrot/maxim_TK_NeuralODEs_TD. This action ensures that our manuscript fully complies with PLOS policy and the best practices of open and reproducible science. In addition, as indicated in the original submission, the original study reports can be requested via email, in case further information is required.

Reviewer #1

This manuscript presents the use of a hybrid TKTD (here GUTS) and NN model to study the effects of mixture on the survival of test organisms. I found the manuscript very well written and highly relevant for the field of mechanistic modelling, ecotoxicology, and environmental risk assessment. I would recommend the manuscript for publication provided minor revisions.

Thank you for your positive assessment and for the detailed, constructive feedback you provided which helped us to improve the manuscript.

Section 2.2.3 The authors used the individual tolerance version of the GUTS model. I would appreciate a reasoning on why this was preferred to the stochastic death one. And if the stochastic death model could be used, what would be the expected differences in performance and predictability?

It is a valid question regarding our choice of the GUTS-IT framework. Our decision was based on both scientific and pragmatic considerations, which we have now clarified in the revised Methods section.

The conceptual difference between the GUTS-IT and GUTS-SD models is most evident under time-variable or pulsed exposure scenarios. In GUTS-IT, mortality is deterministic once an individual tolerance threshold is exceeded; in GUTS-SD, exceeding the threshold initiates a stochastic process of mortality. For the specific experimental context of our study (acute 96-hour toxicity tests with constant exposure concentrations) the literature suggests that the two models often yield very similar survival predictions and goodness-of-fit statistics (see our publication Baudrot and Charles, 2019). As our current dataset does not include the kind of dynamic exposures that would differentiate between the two frameworks, GUTS-IT provided a robust and sufficient basis for our investigation. Since the focus was on testing the performance of the NN approach rather than the original GUTS framework, we believe that testing one of the GUTS variants (SD) is sufficient and adding another variant (IT) will not lead to additional insights.

Additionally, our study involved a substantial and intricate comparison of eight distinct TKTD-bridge architectures across 99 datasets, resulting in 792 calibrations ; and also predictions for 50 chemical pairs, that is 400 simulation-based predictions. Introducing a GUTS-SD variant for each of these eight architectures would have doubled the number of models. This would have significantly increased the complexity of the analysis and potentially obscured our primary research question: to evaluate the performance of the bridge component (linear vs. NN) rather than the toxicodynamic component (IT vs. SD).

Section 2.6. Please explain how the calibration was performed and which criteria were used to assess convergence?

We apologize for omitting these crucial details. As we explained in our response to the Editor, we have created a new section. In section 2.4 Implementation, we added the following paragraph:
L.303-L.308 “*For the MCMC configuration, we ran three chains, each with 7 500 iterations (5 000 for warm-up phase and 2 500 for chain convergence). We checked the convergence of the MCMC using the Gelman-Rubin diagnostic (R<1.05 for all parameters), ensuring an Effective Sample Size (ESS) of*

over 400 to guarantee stable estimation of the posterior distribution. We also checked the trace to confirm good mixing and the absence of trends.”

Section 2.9. Were the predictions performed with constant exposure only or also with peak or with e.g. FOCUS time-windows? Please precise. If not already performed, some example of predictions with variable exposure would also be appreciated.

It is correct. Our predictions were performed under constant exposure profiles that were consistent with the calibration dataset. This has now been made this explicit in the revised text. We fully agree that applying and validating the model under more realistic, time-variable exposure scenarios is a critical next step for its application in environmental risk assessment. As mentioned in our response regarding the GUTS-IT/SD choice, we have expanded the Discussion section to clearly state that the focus on constant exposure is a limitation of the current study, and to emphasize that investigating pulsed and time-variable exposures is a high-priority area for future research. See our comment on the Discussion section.

Fig 4. Please explain in the caption or in the text the scales used on this figure and their interpretation. e.g. How different are NRMSE values of 0.006 and 0.011 or WAIC values of ca 0 and 3 (panel dataset:add)?

Yes, it’s important to include some information on it. NRMSE and WAIC do not have strict absolute scales and should be interpreted by comparing models that have been calibrated on the same dataset. Their formulas are defined by equations 12 and 14 of the manuscript, respectively. For instance, an NRMSE of 0.006 indicates an error approximately 1.8 times smaller than an NRMSE of 0.011. Regarding WAIC, which measures a model deviance, it is commonly accepted that a WAIC difference less than 2 is considered negligible; that a difference between 2 and 7 indicates a weak error; and that a difference greater than 10 suggests a strong error. In the manuscript, we changed section 2.5 about goodness of fit accordingly:

L.333-L.342: *“It is important to note that both NRMSE and WAIC are relative metrics. Their absolute values are not meaningful in isolation; instead, they are used to compare the performance of different models that have been calibrated on the same dataset. For NRMSE, a model with an NRMSE of 0.02 has a root mean square error twice as large as a model with an NRMSE of 0.01, indicating a comparatively poorer fit to the data. Regarding WAIC, estimating predictive accuracy while penalizing for complexity, it is commonly accepted that a difference between models of less than 2 is considered negligible, while a difference of more 10 suggests a strong error.”*

Section 3.3.1 "this deviation metric ranges from 0 to 1". Wouldn't it be from -1 to 1 here?

Indeed. It was an error. Corrected.

Discussion:
All of the used datasets used constant concentrations over time. As variable and pulse exposures are more realistic and relevant for risk assessment, could the authors indicate if this was considered and discuss what would be the expected effects on the performance and predictability of the model?
A discussion point on recommendations for practical use, maybe in perspective with the current approaches would be appreciated.

Yes, as already mentioned, our predictions were performed under constant exposure profiles, consistent with the calibration and validation dataset, but limiting the application on realistic scenarios. We added this point in the discussion:

L.598-L.604: *"A key limitation of the present study is that our model validation was exclusively performed using data from standard acute toxicity tests with constant exposure concentrations only. While this aligns with the calibration dataset, environmental exposures are typically variable, occurring as dynamic pulses over time. Therefore, a high priority for future research is to validate the predictive capabilities of our modelling approach against datasets featuring such time-variable exposure profiles."*

L583 - 587 and 588 - 595 seem to be a repeat/rephrasing of each others. Please check.

Yes, thank you, we have reviewed the indicated lines.

Conclusion:
I would appreciate a point in the conclusion on how "ready" do the authors see their model for practical use and which next steps are identified for further research.

We have revised the conclusion to address this directly.

L.679-L.684: *"In conclusion, while our hybrid model has proven to be a powerful and promising research tool, demonstrating its utility with standard toxicological data, its transition to a framework ready for widespread regulatory application is contingent upon future validation against datasets featuring time-variable exposures and more pronounced non-additive interactions. We identify this as the critical next step for this research."*

Reviewer #2

The manuscript presents a Bayesian neural-ODE extension of the GUTS TKTD framework to model survival of fish exposed to pesticide mixtures. Integrating a mechanistic TK/TD core with a neural bridge is, in principle, timely and could move mixture toxicology beyond purely additive assumptions. However, several aspects of study design, model justification, transparency, and presentation currently limit the manuscript's impact and reproducibility.

Thank you very much. We are very grateful for this rigorous and detailed review, which has prompted significant improvements to the transparency of the manuscript and its scientific outcomes.

1. The paper states that "all scripts and data are available in the Supplementary Material" yet the empirical study reports can only be obtained via e-mail request to Bayer. Public repositories (e.g. Zenodo, GitHub) with open licences should host all raw survival counts, exposure concentrations, and analysis code to enable independent reproduction.

Yes, the link was omitted from the initial submission. However, we have created a public GitHub repository containing all the materials required for the full and independent reproduction of our study. The repository can be accessed at:
https://github.com/virgile-baudrot/maxim_TK_NeuralODEs_TD.

This repository includes: all raw data files, containing survival counts and exposure concentrations for all 99 studies; commented R scripts to perform all model calibrations and validations; scripts to generate every figure and table presented in the manuscript. The Data Availability Statement in the manuscript has been updated to guide readers to this repository, ensuring compliance with PLOS policy and open science best practice.

As mentioned earlier, the study reports can be requested in case any information beyond the material available from the GitHub repository is required.

2. The authors conclude that "most compound combinations adhered to CA or IA; the added value of neural networks is therefore low" (lines 483-491). Yet the manuscript still promotes the NN as the default choice.

This is a well-founded critique. Indeed, the revised manuscript no longer advocates the NN model as the default option. Instead, the simpler linear-bridge model is now positioned as the parsimonious, robust, and interpretable baseline, and is the preferred option for regulatory applications, especially given that most mixtures conform to additivity assumptions.

The NN model is now presented as a powerful, albeit more complex and data-intensive exploratory tool. It should be used selectively, either when there is strong prior toxicological reason to suspect non-linear interactions (such as synergism or antagonism) or when model selection criteria (such as a significantly lower WAIC or NRMSE on cross-validation data) provide strong evidence that the additional complexity is warranted. This revised framing aligns our conclusions directly with our empirical results and provides more responsible guidance for potential users of this framework.

In the Abstract we changed: "*Our findings highlight the potential of combining mechanistic models with machine learning techniques to advance predictive accuracy in environmental toxicology, providing a framework to anticipate the combined effects of untested chemical mixtures and inform risk assessment practices.*" to "*Our findings demonstrate that while simpler linear models provide a robust and parsimonious baseline for predicting additive mixture effects, the neural network component serves as a powerful tool for selectively identifying and forecasting significant non-linear deviations from these expectations. This dual approach clarifies the distinct roles of both simple and complex models, providing a more responsible framework to anticipate the combined effects of untested chemical mixtures and inform risk assessment practices.*"

At the beginning of the Discussion section, we added:
L.509-L.520: "*A central finding of this study is a more nuanced understanding of the respective roles of linear and neural network-based models in mixture toxicology. Our results challenge the notion that a more complex NN model should be the default choice. Instead, we propose that the simpler, linear-bridge model should be considered the parsimonious and interpretable baseline. This model allows for sufficient and more robust predictions for effects of novel mixtures that adhere to additivity assumption, which is critical for regulatory applications. For now, the NN-based model is best framed as a powerful, data-intensive exploratory tool, for example when prior toxicological knowledge suggests non-linear interactions, or when model selection criteria (e.g., WAIC or NRMSE) provide strong quantitative evidence that the additional complexity is warranted by the data.*"

This is also mentioned in the concluding sentence, as already noticed earlier in our response to reviewer #1.

3. Provide a quantitative comparison (e.g. ΔWAIC, NRMSE) between the best linear model and the best NN on an external test set, not only on cross-validation folds. If predictive gains are negligible, a simpler linear bridge may be preferable for regulatory use.

We agree that a direct quantitative comparison on an external test set is essential for robust model evaluation. We would like to clarify that our "Validation" approach, which involves splitting the data into a calibration set and a validation set (either by random time-point selection, or by time-series; see Supplementary material), was designed to serve precisely this purpose. By training the model on a dataset that contains no specific mixture, and then testing it on the held-out pair, we are effectively using an external test set to evaluate the model's ability to generalize to entirely novel combinations. This is the purpose of Figures 5 and 6.

Figure 6, as well as the surrounding text, shows that, for the majority of novel mixtures in our dataset, the simpler, more parsimonious linear model provides more accurate predictions. This directly addresses the reviewer's point that a simpler bridge may be preferable for regulatory use, and we now present this as a key conclusion in our paper. We believe that this revised presentation fully addresses the reviewer's concern by providing the requested quantitative comparison on what is effectively an external test set.

4. Explain why additional regularization (e.g. weight decay, Bayesian hierarchical shrinkage) or simpler priors were not applied given the over-fitting noted by the authors themselves (lines 506-510).

In a Bayesian context, specifying a prior is a form of regularization itself, as it prevents the weights from tending towards infinity. Specifically, applying L2 weight decay is mathematically equivalent to placing a zero-centered Gaussian (Normal) prior on the model weights. We apologize for not making this clear in the original submission, as we failed to document the priors used for the neural network parameters (but we fixed this point). For the record, our initial models did employ a regularizing prior on all weights and biases, specified as a Normal distribution with mean=0 and precision=4 in our JAGS implementation. This corresponds to a standard deviation of 0.5, which already penalizes large weights. This information, along with all the other priors, is now fully detailed in the new Table 2 (see our answer to question 7).

Following the pertinent suggestion, we acknowledge that our initial priors were likely too uninformative, which may have led to the observed overfitting. Also, using Bayesian inference is often subject to criticism for relying on overly informative priors, which can unduly influence the model. For this model presentation, we did not wish to raise that debate. We are also grateful for the suggestion to explore Bayesian hierarchical shrinkage. While implementing such a model was beyond the scope of this revision, we agree that it is a powerful technique and have highlighted it as a key area for future research. We added a sentence to acknowledge this in the manuscript.

L.539-L.542: "*Our initial priors were likely too uninformative, but using Bayesian inference is often subject to criticism for relying on overly informative priors, which can unduly influence the model. For this model study, we did not wish to raise that debate.*"

5. Figure 6 shows that when entire binary mixtures are removed from training, linear models often predict better than NN models (lines 405-409). This undermines the claim that the NN generalizes to novel mixtures.

Correct. Our original claim about the NN ability to generalize to novel mixtures was overstated and not fully supported by our results as presented in Figure 6. This has been corrected this in the revised manuscript. As mentioned in our response to your question 2, the abstract, discussion and conclusion have been substantially revised to emphasize that linear models often perform better than NN models. Accordingly, the linear-bridge model is now presented as the parsimonious and interpretable baseline in order to retract the claim that the NN generalizes to novel mixtures.

6. Suggest reporting separate performance metrics for: (a) new time points for known mixtures, (b) new concentration ratios, and (c) new ingredient combinations—highlighting where the model does or does not extrapolate.

Suggestion (a), which involves predicting new time points for known mixtures, is addressed by our random time-point and time-series cross-validation (Figure 5). Suggestion (c), which involves predicting new ingredient combinations, is addressed by our leave-one-pair-out validation (Figure 6). Suggestion (b), which involves predicting new concentration ratios, is covered by the prediction method, but we agree that it is not distinct from suggestion (a) which involves predicting new time points. The problem is that we cannot make this distinction in terms of the performance metrics.

7.Priors, sampling settings, and convergence diagnostics are not reported. Without them it is impossible to judge parameter identifiability, especially for deeper NNs with many weights. Include a full prior table, sampler settings, and trace/diagnostic summaries in the supplement.

We fully agree. A new section has been added to the manuscript, which includes a description of the MCMC and convergence diagnostics, as well as a table of prior hyperparameters (see the new Table 2 in the manuscript). The script, code and data for the parameters are also provided in the supplementary material and the Github repository.

8.Several paragraphs in Sections 4.4–4.5 repeat sentences almost verbatim (e.g. lines 581-595). Please streamline to improve readability.

Correct. This point was also raised by reviewer #1. We made the necessary changes.

Minor comments:
1. Clarify the use of “damage addition” versus “concentration addition” and ensure consistent notation in equations (sub- and superscripts).

It is true that “damage addition” differs from the foundational concept of “concentration addition”. We clarified this in the introduction:

“In this approach [Damage Addition], the internal damage of each substance is combined additively to provide input to the TD component of the framework. The underlying assumption is that, although substances may have independent TK pathways, their internal effects ultimately converge and accumulate additively within a common TD pathway. This represents an intermediate hypothesis between CA, which assumes a similar mode of action in both the TK and TD, and IA, which assumes

that TK and TD pathways can be completely different between substances. While this additive approach of damage allows predictions to be made across varied exposure profiles, it does not capture non-additive interactions such as synergy and antagonism."

2. Figures 4-7 colour bars: Provide perceptually uniform colour maps and add units to axes (e.g. concentration in µg L^{-1}).

Right, the x-axis was missing from Figure 7. It has now been fixed and labelled with units.

3. In the figure, the authors express "Figure" but use "Fig." in the main text.

It is changed according to Plos standard: see guidelines in https://journals.plos.org/plosone/s/figures

"Label. Name figure labels using Arabic numerals, and abbreviate the word "Figure" to "Fig" (e.g., Fig 1, Fig 2, Fig 3, etc.)." and "Cite figures with the format: Fig 1A, Fig 1B, Fig 2, Fig 3, etc."

4. The discussion cites recent PK/PD neural-ODE work, but could also discuss contemporary ecotoxicological mixture-omics approaches that leverage self-supervised learning.

The literature on machine learning in PK/PD modeling is expanding rapidly. We are grateful to the reviewer for highlighting the advantages of using mixture-omics approaches in ecotoxicology, and for emphasizing that integrating machine learning into TK/TD models, as we have done, could facilitate connections with other datasets, such as mixture-omics. Although we attempted to include such a reference in the manuscript, our discussion section is already quite extensive. Adding this point could cause confusion and distract from our main objective.

Reviewer #3

The manuscript "A Bayesian Neural Ordinary Differential Equations Framework to Study the Effects of Chemical Mixtures on Survival" introduce a new methodology that integrates TKTD models using Ordinary Differential Equations (ODE) with neural networks (NN), offering a robust framework for modeling complex substance interactions. Then this hybrid model was evaluated across 99 acute toxicity studies that included various PPP mixtures, testing its ability to identify and forecast deviations from expected mixture behaviors. This research highlight the potential of combining mechanistic models with machine learning techniques to advance predictive accuracy in environmental toxicology. Generally, I think this an interesting work with novel method in computational toxicology.

Thank you. It is truly motivating to have our work on integrating TKTD models with neural networks, and the robustness of our hybrid approach recognised as novel and potentially impactful.

Several suggestions

1. More details on the experiments settings should be provided, at least in SI.

We agree. A new section has been added to the Supplementary Information providing more detailed information on the experimental settings. This includes specifics of the OECD 203 guideline, the test organism (rainbow trout), water quality parameters, and observation schedules. The aim is to give the reader a fuller context for the data used.

2. For the Fig. 7, what does the x axis mean?

Yes, it was missing. The axis has been correctly labelled "deltamethrin (µg/L)" in the revised figure.

3. GUTS-SD equation should be presented in the main text.

As we only used the GUTS-IT and not the GUTS-SD, we do not think it is valuable to include the latter in the manuscript. We have also added an argument to the Methods section justifying our use of GUTS-IT rather than GUTS-SD, as requested by reviewer #1.

4. As there are many parameters (except NN model) in the model, it is necessary to state clearly how these parameters are estimated. I suggest these estimated parameters could be listed in a supplementary table.

Yes, it's totally correct; In the first version, we have not given this crucial information. As we explained in our response to the Editor and review #2, we have created a new section. In section 2.4 Implementation, we added the following paragraph:

L.303-L.308 *"For the MCMC configuration, we ran three chains, each with 7 500 iterations (5 000 for warm-up phase and 2 500 for chain convergence). We checked the convergence of the MCMC using the Gelman-Rubin diagnostic (R<1.05 for all parameters), ensuring an Effective Sample Size (ESS) of over 400 to guarantee stable estimation of the posterior distribution. We also checked the trace to confirm good mixing and the absence of trends."*

Also, now, all raw survival data, exposure concentrations, and the complete R analysis scripts required to reproduce every result, figure, and table in the manuscript are now publicly available without restriction in a dedicated GitHub repository. This repository can be accessed at:

https://github.com/virgile-baudrot/maxim_TK_NeuralODEs_TD. This action ensures that our manuscript fully complies with PLOS policy and the best practices of open and reproducible science. In addition, as indicated in the original submission, the original study reports can be requested via email, in case further information is required.

Reviewer #4

This study presents a novel modeling framework for predicting mixture toxicity, integrating neural networks with toxicokinetic–toxicodynamic modeling. The manuscript is well-structured, clearly written, methodologically sound, and provides valuable insights into both model development and toxicological interpretation. The graphical presentations are generally effective, and the discussion is thorough and informative. In my view, the manuscript is almost publishable as is, but I offer the following comments for the authors to consider in their revision for better clarity.

We would like to thank you for your positive and constructive feedback on our study. We are delighted that you recognize the clarity of its structure, its methodological rigor, and its scientific value.

Figure 4: Consider replacing point shapes with circled numbers (e.g., ①②③) to make model distinctions more readable. However, this is a non-essential stylistic suggestion.

Thank you for your suggestion. As other reviewers mentioned, some information was missing, making it difficult to understand. We have therefore followed their suggestions.

Figure 5: The caption claims that point shape indicates cross-validation type (time-points vs time-series), but the legend uses shape for model type. Check to ensure consistency.

Done. Indeed, it was an error.

Equation 14: Use roman font for "WAIC".

Done.

Line 407, Figure 6: Should "bar(s)" be "points"? Figure 6 uses points, not bars.

Indeed. It was not "Horizontal bars" but "x-axis".

Figure 7: The x-axis title is missing.

Right. Fixed.

Figure 8: (a) Is the "Mean Penalized Deviation" here the same as "deviation" in Equation 11? Use consistent terminology to avoid possible confusion. (b) If no observed data exist for red-font mixtures, how was the deviation (as defined in Eq. 11) computed? (c) I suggest revising "Missing point appear when..." to "Missing points are due to the 95% credible interval spanning more than 50% of the survival probability range."

(a) Yes, we changed the name of equation 11 to match with the terminology of Figure 8. MPD stands for Mean Penalized Equation. The change was made on line 274 (before equation 11) and in the caption of Figure 8.
(b) As we mentioned after equation 11, "the term 'observed_i' refers to the value calculated by our model, while 'predicted_i' corresponds to the expected value under the CA or IA hypotheses". Consequently, real data is not required. What we need is a prediction by our model compared to what it would be under the IA and CA hypotheses. However, you are right that "observed" is misleading, so we have changed "predicted" to "benchmark" and "predicted" to "observed". So, we now have the following sentence: "the term 'predicted_i' refers to the value calculated by our model, while 'benchmark_i' corresponds to the expected value under the CA or IA hypotheses".
(c) Ok, suggestion accepted.

Lines 448-450: While excluding high-uncertainty data is acceptable for interpretation, showing those data could highlight important research gaps and possible synergistic effects.

We did not exclude any experimental data, but we did omit predictions associated with very high uncertainty. Since we summarize each prediction using only the "mean deviation" value, including predictions with substantial uncertainty could distort the interpretation of the results. Note, we used the range to represent uncertainty. Removing the mean deviation point indicates that the model was unable to make accurate predictions. We added the following sentence: "This step was necessary because we summarize each prediction using a single "mean deviation" value. Including points with

excessively large uncertainty could distort the interpretation of the overall results. Removing a point indicates that the model failed to provide a reliable prediction."

Lines 451-452: The sentence "...each line on the graphs represents one of the pairs tested" is unclear. What does the "line" refer to? Horizontal or vertical lines? What each "pair" represents? Chemical pairs? Model-deviation pairs?

Right. It's a mistake; we have to read "each row" and "each pair of chemical product".

Have the authors made all data and (if applicable) computational code underlying the findings in their manuscript fully available?

The PLOS Data policy requires authors to make all data and code underlying the findings described in their manuscript fully available without restriction, with rare exception (please refer to the Data Availability Statement in the manuscript PDF file). The data and code should be provided as part of the manuscript or its supporting information, or deposited to a public repository. For example, in addition to summary statistics, the data points behind means, medians and variance measures should be available. If there are restrictions on publicly sharing data or code —e.g. participant privacy or use of data from a third party—those must be specified.

Reviewer #1: Yes

Reviewer #2: **No:** The empirical fish-survival data are proprietary and only available on request from Bayer. This conflicts with open-science best practices and PLOS policy. All raw survival counts, exposure profiles, and analysis scripts should be deposited in a public repository (e.g., GitHub / Zenodo)

Reviewer #3: Yes

Reviewer #4: Yes

PLOS authors have the option to publish the peer review history of their article (what does this mean?). If published, this will include your full peer review and any attached files.

If you choose "no", your identity will remain anonymous but your review may still be made public.

Do you want your identity to be public for this peer review? For information about this choice, including consent withdrawal, please see our Privacy Policy.

Reviewer #1: No

Reviewer #2: **Yes:** Wei-Chun Chou

Reviewer #3: No

Reviewer #4: **Yes:** Tan, Qiao-Guo

[NOTE: If reviewer comments were submitted as an attachment file, they will be attached to this email and accessible via the submission site. Please log into your account, locate the manuscript record, and check for the action link "View Attachments". If this link does not appear, there are no attachment files.]

Figure resubmission:

While revising your submission, please upload your figure files to the Preflight Analysis and Conversion Engine (PACE) digital diagnostic tool, https://pacev2.apexcovantage.com/. PACE helps ensure that figures meet PLOS requirements. To use PACE, you must first register as a user. Registration is free. Then, login and navigate to the UPLOAD tab, where you will find detailed instructions on how to use the tool. If you encounter any issues or have any questions when using PACE, please email PLOS at figures@plos.org. Please note that Supporting Information files do not need this step. If there are other versions of figure files still present in your submission file inventory at resubmission, please replace them with the PACE-processed versions.

Reproducibility:

To enhance the reproducibility of your results, we recommend that authors of applicable studies deposit laboratory protocols in protocols.io, where a protocol can be assigned its own identifier (DOI) such that it can be cited independently in the future. Additionally, PLOS ONE offers an option to publish peer-reviewed clinical study protocols. Read more information on sharing protocols at https://plos.org/protocols?utm_medium=editorial-email&utm_source=authorletters&utm_campaign=protocols
