# Supplementary material for: A Bayesian neural ordinary differential equations framework to study the effects of chemical mixtures on survival
Source: PLoS Comput Biol. 2025 Nov 10;21(11):e1013681. doi: 10.1371/journal.pcbi.1013681 (PMC12674509; doi:10.1371/journal.pcbi.1013681)
Supplement: Attachment — Submitted filename: Response to Reviewers PCOMPBIOL-D-25-00616R1 _ 2ndRUN (1).pdf [file pcbi.1013681.s005.pdf]

Virgile BAUDROT
Qonfluens SAS, Montpellier
virgile.baudrot@qonfluens.com

Editor
PLOS Computational Biology

Dear Editor,

Thank you for handling the review of our manuscript. We sincerely appreciate the positive evaluations provided by the reviewers on this revised version and are grateful for the recommendation to accept the paper.

We have carefully considered and addressed the two points raised by Reviewer #2. Below, we provide a detailed, point-by-point response outlining the corresponding changes made in the manuscript.

Sincerely,
Virgile Baudrot
on behalf of all authors

Reviewer's Responses to Questions

Comments to the Authors:
Please note here if the review is uploaded as an attachment.

Reviewer #2: I appreciate the authors' comprehensive and thoughtful revisions. The manuscript is now much clearer, and the framing of the linear versus neural models is balanced and responsible. Most of my original comments have been fully addressed. I have only two minor suggestions for further clarity:

1. Figures 5–6 nicely illustrate the model performance, but it would be helpful to summarize key quantitative metrics (for example, ΔWAIC or NRMSE values) comparing the linear and NN models in a concise table or sentence in the Results section. This would make the relative performance difference immediately visible to readers.
We added a table summarizing statistics on NRMSE values for Figure 6 with the following sentence in the text in results: "*Summary statistics from Table 5 show that, on average (both in terms of mean and median), the linear model performs better than models that include a Neural Network.*"
For table 5, we just added those statistics in the text: "*For the approach using time-points, in mean (standard deviation) the NRMSE are respectively for no-split and split linear models of 0.877 (0.0887), 0.912 (0.0816) models and for no-split and split NN models of 0.918 (0.0857), 0.876 (0.0813) ; and for*

the approach on the time-series, in mean the NRMSE are respectively of 0.400 (0.0814), 0.409 (0.0856) for linear models and 0.419 (0.0905), 0.422 (0.0907) for models with NN."

2. Please briefly clarify why new concentration-ratio predictions cannot be separated from new time-point predictions in your validation framework, so that readers understand this methodological constraint.

In fact, we could have separated these two types of predictions: for the "prediction on reformulation", by selecting only the time series so that the concentration ratios in the validation data would not appear in the training data, and for the "prediction at new time", by removing data points corresponding to specific time steps from the training set.
However, although this would certainly be of interest, the key point is that in the subsequent predictive phase (without validation data), time and concentration are dynamically coupled through the ODE-based toxicokinetic model. A new concentration ratio necessarily produces a new temporal trajectory of internal concentrations and damages. Therefore, it is not possible to isolate new concentration-ratio predictions from new time-point predictions, as both dimensions are intertwined in the structure of the model.
We added this comment in the manuscript:
L.354-360: "*We could have designed the training and validation subsets in a more structured way, for example to specifically test predictions on reformulation or predictions at new time points (see Supplementary Material). However, since time and concentration are dynamically coupled through the ODE-based toxicokinetic model, a new concentration ratio inherently generates a new temporal trajectory. As a result, these two types of predictions cannot be evaluated independently."*

With these small clarifications, I consider the manuscript fully responsive to prior comments and suitable for publication.

We thank Reviewer #2 for the helpful comments and are glad that the revised manuscript is now considered suitable for publication.

Reviewer #3: My comments are well addressed. The paper can be accepted in the present form.

We sincerely appreciate the positive evaluation of Reviewer #3 and are grateful for the recommendation to accept the paper in its current form.
